# Evidence of Subdivisions on Evolutionary Timescales in a Large, Declining Marsupial Distributed across a Phylogeographic Barrier

**DOI:** 10.1371/journal.pone.0162789

**Published:** 2016-10-12

**Authors:** Deryn L. Alpers, Faith M. Walker, Andrea C. Taylor, Paul Sunnucks, Steven Bellman, Birgita D. Hansen, William B. Sherwin

**Affiliations:** 1 School of Veterinary and Life Sciences, Murdoch University, Murdoch, Western Australia, Australia; 2 School of Biological Sciences, Monash University, Clayton, Victoria, Australia; 3 University of South Australia, Adelaide, South Australia, Australia; 4 School of Forestry, Northern Arizona University, Flagstaff, Arizona, United States of America; 5 University of New South Wales, Kensington, New South Wales, Australia; 6 Centre for eResearch and Digital Innovation, Federation University Australia, Ballarat, Victoria, Australia; 7 Murdoch University Cetacean Research Unit, Murdoch University, Murdoch, Western Australia, Australia; Universita degli Studi della Tuscia, ITALY

## Abstract

Major prehistoric forces, such as the climatic shifts of the Pleistocene, can remain visible in a species’ population genetics. Inference of refuges via genetic tools is useful for conservation management as it can identify populations whose preservation may help retain a species’ adaptive potential. Such investigation is needed for Australia’s southern hairy-nosed wombat (*Lasiorhinus latifrons*), whose conservation status has recently deteriorated, and whose phylogeographic history during the Pleistocene may be atypical compared to other species. Its contemporary range spans approximately 2000 km of diverse habitat on either side of the Spencer Gulf, which was a land bridge during periods of Pleistocene aridity that may have allowed for migration circumventing the arid Eyrean barrier. We sampled from animals in nearly all known sites within the species’ current distribution, mainly using non-invasive methods, and employed nuclear and mitochondrial DNA analyses to assess alternative scenarios for Pleistocene impacts on population structure. We found evidence for mildly differentiated populations at the range extremes on either side of Spencer Gulf, with secondary contact between locations neighbouring each side of the barrier. These extreme western and eastern regions, and four other regions in between, were genetically distinct in genotypic clustering analyses. Estimates indicate modest, but complex gene flow patterns among some of these regions, in some cases possibly restricted for several thousand years. Prior to this study there was little information to aid risk assessment and prioritization of conservation interventions facilitating gene flow among populations of this species. The contributions of this study to that issue are outlined.

## Introduction

The Pleistocene period was marked by oscillations in global climate aridity and, as a consequence, habitat fragmentation with animal species potentially surviving in refuges [1, 2]. Descendants from different Pleistocene refuges may carry valuable genetic diversity that can be identified by well-established population genetic approaches [3–5]. Many species exhibit genetic effects indicative of Pleistocene refuges, including most Australian species [2, 6–8], but some Australian fauna appear to have responded idiosyncratically to Pleistocene climatic fluctuations, ranging widely rather than being confined to refuges [9].

**Fig 1:**
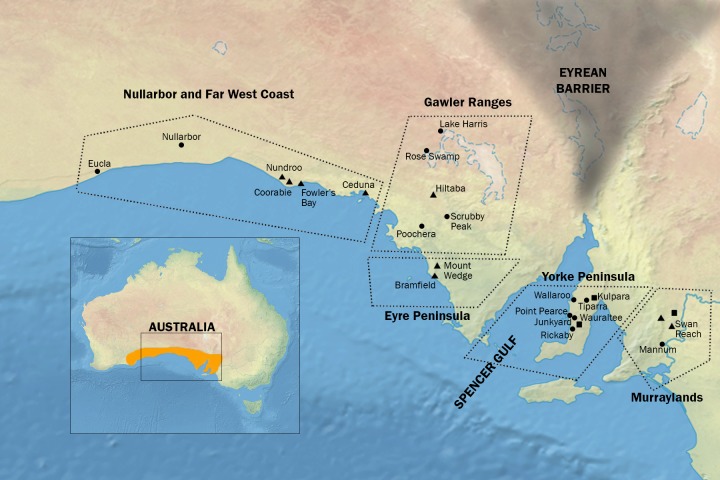
Collection Sites. Southern Australian collection sites for the *Lasiorhinus latifrons* (southern hairy-nosed wombat) samples used in this study. ▲ = Set A, ● = Set B, ■ = Sets A and B. Dotted lines indicate the major geographic regions where these sites were located. Inset shows probable species distribution of *L*. *latifrons* prior to European settlement and the main map shows the hypothesised extent of the Eyrean Barrier in the late Pleistocene, which could have restricted gene flow between east and west. Map from Natural Earth (public domain): http://www.naturalearthdata.com/.

Irrespective of historical processes, conservation management policies can use information on population structure to maximize the likely retention of a species’ evolutionary history and adaptive diversity by minimizing the loss of distinct populations. These conservation management concerns are pertinent for the southern hairy-nosed (SHN) wombat (*Lasiorhinus latifrons*), a large, herbivorous, fossorial marsupial from semi-arid regions of southern Australia (Fig 1) [10, 11]. Currently the SHN wombat has a fragmented distribution, with relatively large populations in areas of sparse human settlement, such as the far west coast and Nullarbor Plain, and smaller remnant populations where agriculture has been intense, such as on the Yorke Peninsula (Fig 1). Fossil evidence suggests that prior to the European settlement of Australia, approximately 200 years ago, the species had a more continuous distribution, and was more widespread (Fig 1 inset, [12, 13]). The SHN wombat’s contemporary range spans the Spencer Gulf, a body of water between the Eyre and Yorke Peninsulas (Fig 1) that did not exist when sea levels were lower during periods of Pleistocene aridity [14]. Identifying how the SHN wombat responded to historical habitat and climatic changes is important for both our greater understanding of its evolutionary history as well as its contemporary conservation.

The SHN wombat is not currently considered threatened as a species (although its status requires review [15]), but under South Australia (SA)’s *National Parks and Wildlife Act 1972*, the Yorke Peninsula populations have Endangered conservation status owing to their small size and isolation due to recent farming activity [16]. In addition, the Murraylands and Lake Acraman (near Hiltaba in Fig 1) populations are considered Vulnerable [13, 17]. In two bioregions (the Nullarbor Plain and the Eyre-Yorke Block) the species is classified as Persisting (present in >50% of its former range), but in the Gawler region (also near Hiltaba in Fig 1) it is classified as Declined (present in 10–50% of its former range), and in the Murray Darling Depression it is classified as Severely Declined (present in <10%) [18]. The most recent estimates of population sizes range from around 10 individuals at some sites on the Yorke Peninsula to over 150 000 in the far west coast (FWC) of SA between Ceduna and the Nullarbor Plain, although even this largest population appears to be currently contracting [13, 19]. Thus many populations are expected to have declining effective population size and reduced inter-population gene flow. Populations under such conditions may experience genetic erosion and inbreeding depression, which limits future adaptation ability and elevates extinction risk [20–22]. These negative impacts are potentially reversible by appropriate facilitated gene flow, so it is important to understand population genetic structure in species of conservation concern [23].

The palaeoecological history of the region inhabited by SHN wombats raises some expectations for patterns in their genetics. Over 70% of Australia is currently arid, but during the Pleistocene period these areas were even drier as water was locked up in ice caps, and many species in Australia were presented with waterless deserts as obstructions to gene flow between populations [2, 24]. One of these was the Eyrean barrier, situated towards the eastern end of the current SHN wombat distribution, and believed to have peaked in impact during the last severe arid period, as recently as 18 000 to 16 000 years ago [25]. The Eyrean barrier drove speciation and subspeciation in semi-arid Australian birds [6, 25–29]. At the western end of the SHN wombat’s distribution, the Nullarbor barrier had similar sub-structuring effects during Pleistocene times on mesic birds, other semi-arid vertebrates, and plants [9]. But for other species, lower sea levels caused by Pleistocene aridity opened up opportunities for dispersal and gene flow. Spencer Gulf, which bisects the current range of the SHN wombat, was a dry land bridge during these arid times [14]. When the Gulf was re-flooded, especially during the minor pluvial period 8 000 to 5 000 years before present, this land bridge would have closed, leading to contraction and isolation of population units. The contemporary distribution of the SHN wombat is patchy (http://maps.iucnredlist.org/map.html?id=40555), but prior to European settlement there may have been a narrow habitat conduit around the northern end of Spencer Gulf (Fig 1 inset [12, 13]). If oscillations in Pleistocene aridity disrupted gene flow in the SHN wombat, contemporary populations could encompass more than one unit of conservation concern, separated in evolutionary time (i.e. evolutionarily significant units [30, 31]). Earlier research based on microsatellite data from four of the 24 populations examined in this study suggested that there is some separation between eastern and western populations of the species [32]. It was clear from this preliminary work that to further investigate this finding across the entire species, more populations needed to be sampled, especially centrally located populations on the Yorke Peninsula, and that future research should investigate gene flow. The present study responded to these needs by increasing the number of populations sampled from four to 24, and investigated whether the east–west separation previously reported was due to expansion from a single refugium on one side of Spencer Gulf, or multiple refugia. This research also investigated to what extent gene flow has been achieved by any dispersal made possible by the Spencer Gulf land bridge, or the narrow conduit to its north that existed until recently.

Here, we examine genetic patterns to distinguish between four different scenarios: 1) no impact of the disappearance of the land bridge, and so no east or west post-Pleistocene refuges; 2) east and west refuges with no or very little gene flow between them, followed by subsequent secondary contact; 3) east and west refuges with little or no admixture; and 4) post-Pleistocene populations surviving only on one side of Spencer Gulf with subsequent recolonization across the current range. We expect that these competing scenarios will be reflected in mitochondrial DNA (mtDNA) and microsatellite DNA data as follows:

*Scenario 1*: ‘no refuges’ would be supported by low divergence between populations on either side of the Spencer Gulf land bridge. This could be due either to insufficient time post the Spencer Gulf barrier, or ineffectiveness of the Eyrean barrier to prevent movement and gene flow.
*Scenario 2*: ‘multiple refuges with secondary contact’ would be supported by divergence between the descendants of putative refuge populations, with estimates of time of divergence consistent with impact of the re-flooding of the Gulf, and maximal genetic diversity where the genes from the different refuges are admixed, unless population size reduction and genetic drift cause loss of variation at the contact zones.
*Scenario 3*: ‘multiple refuges without admixture’ also predicts divergence between the refuges, like Scenario 2, but does not predict higher diversity in between, given no admixture. As small numbers of wombats spread out into the colonised areas, many genotypes may have survived, but rare alleles may have been lost during successive drift events.
*Scenario 4*: ‘recolonisation from one side of Spencer Gulf’ would be supported by a lack of strong genetic division, and by genetic diversity gradually reducing with increasing distance from the refugium, or homogeneity or isolation-by-distance if gene flow has been sufficient. In this case, genetic subdivision should be unrelated to putative phylogeographic barriers and times of high aridity when the gulf was dry.

We investigated the effects of environmental influences since the late Pleistocene on SHN wombat population structure, and attempted to uncover the population genetic patterns that existed prior to land-use by European settlers and the relationships among extant populations. We applied microsatellite and mitochondrial DNA data, collected from sites across the entire current range of the SHN wombat, to estimate genetic differentiation and gene flow at different time scales. We provide genetically based recommendations for conservation management of the SHN wombat, building upon previous preliminary studies of the genetics of this species [32–34] and the broader literature [23]. This paper also highlights the use, at very broad geographical scales, of non-invasive genetic data collection techniques for rare or elusive species: data for this paper were obtained mostly from hairs captured on adhesive tape at burrow entrances [34–38].

## Materials and Methods

### Sampling and DNA extraction

This study’s protocol was approved by the Committee on the Ethics of Animal Experiments of the University of New South Wales (Permit Numbers: 93/65 and 94–26). The NSW National Parks and Wildlife Service approved the holding of samples from South Australia in NSW (Permit Numbers: A941 and B1025). All efforts were made to minimize impacts on animals, and so DNA was extracted mainly from hair samples collected on sticky tape from wombats as they entered or left their burrows [34, 36–38]. Blood samples were taken from the cephalic vein of anaesthetised animals. No animals were killed specifically for this study, but tissue samples were obtained opportunistically from road-kill animals or animals killed by farmers under SA Department of Environment and Natural Resources destruction permits (Permit Numbers: S11017 and A02340). Destruction permits require that wombats are killed only by a clear brain shot from a high-powered rifle (minimum calibre .243; 6.2 mm) [39]. Wombats were sampled at 24 sites in five geographic regions (Nullarbor Plain and FWC; Gawler Ranges—the area around Rose Swamp, Lake Harris, Hiltaba, and Scrubby Peak; Eyre Peninsula; Yorke Peninsula; and the Murraylands; Table 1, Fig 1), representing all major and >90% of minor known populations of the entire species [34]. The SA Department of Environment and Natural Resources gave permission for locations in national parks. For locations on private land, the owner of the land gave permission to conduct the study on that site (names of owners are listed in [19] and [40]; available from the authors on request). Samples and data were collected during two separate time periods, 1994–1996 (set A) and 2000–2002 (set B), with the intervening time being unlikely to be genetically relevant given the longevity of the species (>15 years in the wild [10]). There were no differences in allele frequencies between sets A and B at any of their three shared sites (averaging over 4 common loci, Brookfield: A = 5.25, B = 7.00, *P* = 0.250; Kulpara: A = 6.00, B = 6.00, *P* = 1.000; Wauraltee: A = 8.00, B = 6.75; *P* = 0.500; Wilcoxon signed-ranks tests). Set B DNA was extracted via same-day 5% Chelex extractions performed in the field (S1 Text) [41]. Set A included DNA from hair but also from blood and tissue, all extracted using phenol/isoamyl alcohol+chloroform extraction protocols (S1) [42, 43]. Blood samples were taken into tubes containing EDTA, and frozen immediately in liquid nitrogen. Tissue samples were frozen immediately in liquid nitrogen in the field then kept at −80°C, or held at room temperature in absolute ethanol (AnalaR) or dimethyl sulphoxide storage buffer [44].

**Table 1:** Estimates of genetic variation at four microsatellite loci in 24 *Lasiorhinus latifrons* sampling sites from southern Australia.

Site	*Data Set*	*Latitude*	*Longitude*	*n*	*AD*	*AR*	*H*_E_	*F*_IS_	*nPA*
**West of the Eyrean Barrier**									
**Nullarbor + Far west coast (FWC)**									
Eucla	B	31°40′S	128°53′E	9.00	4.50	3.02	0.63	0.00	0
Nullarbor	B	30°51′S	130°28′E	27.00	5.00	2.86	0.62	-0.10	0
Nundroo	A	31°47′S	132°12′E	12.25	7.00	3.83	0.75	0.26	1
Coorabie	A	31°54′S	132°18′E	13.50	6.25	3.66	0.75	0.00	1
Fowler’s Bay	A	31°58′S	132°34′E	6.00	4.50	3.52	0.70	-0.15	0
Ceduna	A	32°08′S	133°41′E	3.00	4.50	4.50	0.72	0.07	0
**Average**				**11.79**	**5.46**	**3.33**	**0.68**	**0.00**	**0.36**
**Gawler Ranges**									
Lake Harris	B	31°04′S	135°14′E	13.00	4.25	2.72	0.51	-0.01	0
Rose Swamp	B	31°17′S	134°55′E	8.75	4.50	3.11	0.64	-0.02	0
Hiltaba	A	32°08′S	135°03′E	14.25	6.75	3.87	0.77	-0.05	2
Scrubby Peak	B	32°31′S	135°19′E	24.00	4.25	2.81	0.59	-0.05	0
**Average**				**15.00**	**4.88**	**3.09**	**0.62**	**-0.04**	**0.48**
**Eyre Peninsula**									
Poochera	B	32°43′S	134°50′E	6.50	4.75	3.50	0.70	0.24	0
Bramfield	A	33°38′S	134°59′E	7.75	4.75	3.21	0.64	-0.01	0
Mount Wedge	A	33°29′S	135°09′E	9.75	3.75	2.70	0.60	-0.15	0
**Average**				**8.00**	**4.34**	**3.08**	**0.64**	**0.00**	**0.00**
**Average (west)**				**11.90**	**5.06**	**3.20**	**0.65**	**-0.02**	**0.35**
**East of the Eyrean Barrier**									
**South and West Yorke Peninsula**									
Rickaby	B	34°43′S	137°31′E	5.00	4.25	3.31	0.63	0.14	0
Wauraltee	A/B	34°30′S	137°36′E	43.50	10.00	3.51	0.73	0.13	1
Point Pearce	B	34°24′	137°26′E	6.25	3.25	2.59	0.53	-0.29	0
Junkyard	B	34°25′	137°30′E	6.75	3.75	2.88	0.61	-0.04	0
Wallaroo	B	33°56′	137°36′E	8.00	4.75	3.12	0.63	-0.13	0
**Average**				**13.90**	**7.77**	**3.31**	**0.68**	**0.05**	**0.63**
**Northeast Yorke Peninsula**									
Kulpara	A/B	34°04′S	138°02′E	81.25	8.25	3.56	0.76	0.01	0
Tiparra	B	34°06′S	137°54′E	6.50	5.00	3.43	0.65	0.07	1
**Average**				**43.88**	**8.01**	**3.55**	**0.75**	**0.01**	**0.07**
**Murraylands**									
Sturt Highway	A	34°25′S	139°08′E	8.00	5.25	3.72	0.75	-0.17	0
Mannum	B	34°54′S	139°18′E	3.75	3.00	2.73	0.50	0.26	0
Swan Reach	A	34°34′S	139°36′E	82.75	9.25	3.89	0.80	-0.02	1
Brookfield	A/B	34°21′S	139°24′E	115.75	7.50	3.66	0.78	0.03	1
**Average**				**52.56**	**8.02**	**3.74**	**0.78**	**0.01**	**0.94**
**Average (east)**				**33.41**	**7.97**	**3.61**	**0.76**	**0.02**	**0.68**
**Average (overall)**				**21.76**	**7.11**	**3.49**	**0.72**	**0.01**	**0.58**

Average sample size per locus (*n*), Allelic diversity (*AD*), allelic richness (*AR*), expected heterozygosity (*H*_E_), inbreeding coefficient (*F*_IS_), and number of private alleles (*nPA*) are presented for each sample sitesite. Sample-weighted averages are calculated.

### Mitochondrial DNA analyses

Two approaches to analyse mtDNA sequence variation were used: (i) Southern blot RFLP analysis of the whole mtDNA genome and (ii) single-strand conformation polymorphism (SSCP) combined with sequencing of a 400-bp section of cytochrome *b*. Cytochrome *b* is useful when looking for longer-term signatures of isolation owing to its relatively well-studied rate of evolution in mammals [45]. Note that mtDNA analyses could be performed only on populations for which tissue samples were available; insufficient DNA was available from single hair samples also being genotyped for microsatellites.

#### Whole mitochondrial genome RFLP

A preliminary RFLP analysis of the entire SHN wombat mtDNA genome used 22 restriction enzymes, five of which revealed polymorphisms characterising two haplotypes, in four individuals from the Murraylands area [33]. Because DNA was limiting for some samples, only three of these polymorphic enzymes (*Acc* I, *Ava* II and *Hpa* I) were employed for population samples, with the primary aim of identifying the geographic distribution of the two known haplotypes. DNA from 47 tissue samples was used, from seven sites spanning the SHN wombat distribution: three locations in the Murraylands in the eastern species range (10 individuals from Swan Reach, 5 Brookfield, 9 Sturt Highway), three locations from the western (FWC) region (6 Fowler’s Bay, 3 Nundroo, 6 Coorabie, and one location on the Eyre Peninsular on the western side of the Eyrean barrier (8 Mount Wedge) (Fig 1). Restriction digests of 4 μg of genomic DNA were performed overnight in 30 μL reactions, and the digests electrophoresed for 15–18 hours at 50–60 V beside a Lambda/*Hin*d III size marker. Southern transfers were performed in 0.4 M NaOH overnight to nylon membranes (Hybond-N+, Amersham). The mitochondrial DNA used as a probe for the hybridisation was from a dasyurid marsupial, the common dunnart *Sminthopsis crassicaudata* (probe pSMB9, 15 kb of mtDNA cloned into pBR322 by Dr Rory Hope and colleagues at Adelaide University). Detailed descriptions of the hybridisation procedure are given in [40]. Each fragment was scored as present or absent in each individual.

#### Cytochrome *b*

Cytochrome *b* PCR analyses were performed on DNA from 64 tissue samples from the same seven sites used in the RFLP survey (21 Swan Reach, 10 Brookfield, 8 Sturt Highway; 6 Fowler’s Bay, 3 Nundroo, 7 Coorabie, 9 Mount Wedge). These 64 samples included all 47 used for the whole genome RFLP analyses, plus 17 more that had insufficient DNA for RFLP analysis but enough for PCR. Universal primers L14724 and H15149 [46] were used to amplify a 400-bp region of the cytochrome *b* gene. Ten μL PCRs were conducted in the presence of 2.5 mM MgCl_2_, 5 pmoles each primer, 200 μM each dNTP, 1 X PCR reaction buffer (Gibco/BRL) with 0.5 units *Taq* polymerase and 0.05 μl α-dATP^33^ for 30 cycles with an annealing temperature of 50°C. Denatured PCR products from all 64 samples were first screened by SSCP [47] to identify sequence variants. This identified two gel phenotype variants. Twelve individuals (representing six of each gel phenotype, and spanning regions) were sequenced for the entire 400-bp fragment in both directions using standard ABI dye-terminator procedures (ABI Prism 377) with the PCR primers as the sequencing primers. There were no sequence differences among members of the same SSCP phenotype from different populations, giving confidence that sequence variation in the included populations had been well assayed. As is typical for mammalian cytochrome *b*, the sequences aligned perfectly with no insertions or deletions being evident.

#### Mitochondrial DNA analytical methods

Genetic diversity parameters were estimated from the southern blot RFLP and SSCP haplotype data using REAP (Restriction Enzyme Analysis Package, version 4.0 [48]). For the RFLP data, REAP also estimated evolutionary distances (based on pairwise distances), and Arlequin was used to estimate a minimum spanning tree, Fu’s *F*_*S*_ test for population expansion and/or selection [49], and Tajima’s *D* test for expansion, bottlenecks, and other departures from the neutral mutation hypothesis [50]. Arlequin was also used for analyses of molecular variance (amova) that estimated variance among groups (Φ_CT_), among populations within groups (Φ_SC_) and within populations (Φ_ST_).

The extent of geographic heterogeneity in the frequencies of haplotypes was assessed using Monte Carlo simulations executed by REAP, following the method of Roff and Bentzen [51]. For each of the two haplotype frequency matrices (RFLP and cytochrome *b*), 10 000 randomisations were performed for each of three datasets: all seven sites together, the three Murraylands sites together in an eastern group and the four western sites (including Mount Wedge from the Eyre Peninsula) in another group.

### Nuclear DNA analyses

#### Microsatellite screening

Although more markers were available by the time of the second study [34, 36, 37, 52], these were not retrospectively applied to sample set A as little DNA remained from those samples. Further, the goals of the second study and the low DNA amounts in single hairs dictated that not all markers used in the first study were scored in sample set B. Hence, only four microsatellite loci were common to both data sets: Lla54CA, Lla67CA, Lla68CA and Lla71CA [32, 33, 35]; four loci– 3AT, Lla16CA, Lla55A and Lkr107 –were unique to set A (making a total of eight for that set [45]); five loci were unique to set B: Ll2, Lk13, Lk21, Lk23 and Lk37 (making a total of nine for that set [19]). Although sets A and B were analysed several years apart, the same amplification and genotyping procedures were used, and reference individuals from set A were used for sizing control (S1) [52]. At the three sites common to sets A and B, we checked for genotype matches and there were none, implying no animal was sampled in both periods. Rigorous quality control procedures were employed to prevent genotyping errors when identifying individuals from anonymously collected hair. Single hairs only were used for set B [34, 36], but both single and pooled hair samples were used for set A. Pooled samples showing more than two alleles at any locus were identified as mixed, and removed. Given the variability of the loci, the probability of identifying mixed sample as a single individual was extremely low: using eight loci in set A, *P*_ID_ < 0.01 [53], and using nine loci in set B, *P*_ID_ < 5.93 × 10^−6^ [54].

#### Genetic diversity from microsatellite data

Sets A and B were combined for analysis of their four shared loci. Where appropriate, separate analyses were conducted for sets A and B using the eight or nine microsatellite loci available, respectively. Genetic diversity measures within sampling sites, sample size per locus, expected heterozygosity, *F*_IS_ and number of private alleles were obtained using GenAlEx (version 6.5 for Excel 2010 [55]). Allelic diversity and allelic richness were calculated using FSTAT (version 2.9.3 [56]). Deviations of genotype proportions from Hardy-Weinberg equilibrium (HWE) were estimated using the Markov chain exact probability method [57] in genepop (version 4.1 [58]). In each sampling site, linkage disequilibrium (LD) among the loci was evaluated using arlequin (version 3.5.1.3 [59]). Friedman tests were used to test whether expected heterozygosity (*H*_E_) and allelic richness (*AR*) differed among sites. Identification of sites with high or low diversity was conducted by comparisons among all pairs of sites using Wilcoxon paired-samples signed-ranks tests with locus as the pairing factor. As there were only four loci, the maximum possible *P*-value for pairwise tests between sites was 0.07, so these are taken as significant.

For situations where many significance tests were carried out, corrections for multiple tests were applied using the sequential Bonferroni technique [60]. Nonetheless, sequential Bonferroni can mask important effects if applied in a blanket fashion, so individually significant results were examined to identify any individual loci or sites that persistently showed deviation from genetic equilibria.

#### Genetic structure using microsatellite data

We used a Bayesian approach to determine the number of distinct population clusters within the combined four-locus sample sets. The program structure (version 2.3.4 [61, 62]) was used to calculate the probability of the data (*X*), given a hypothesized number (*K*) of clusters, Pr(*X*|*K*), for all possible *K*, from 1 to the total number of collection sites (24 sampling sites, 12 sampled in set A, 16 in set B, with 4 sites in common). Structure is a genotype-based analysis and hence will reflect shorter-term gene flow than will genic measures (i.e. based on allele frequencies). We used the approach of Evanno *et al*. [63] to identify the number of clusters, setting the parameters to their default values, as recommended by the structure users’ manual. After identifying the most likely *K* (which required 20 runs for each of the 24 potential *K*, using a minimal 10 000 burn-period followed by 10 000 replications for each run [63]), a final run was undertaken with a burn-in period of 30 000 followed by 500 000 replications. The groups of sites identified by structure as sharing genetic cluster membership were used to specify the population groupings for analyses of molecular variance (amova), carried out using Arlequin, to quantify genetic differentiation in a framework based on allele proportions.

Differences in allele proportions between samples from different sites were examined using GenAlEx, which was also used to calculate the mutual information index, ^*S*^*H*_*UA*_, which provides a more robust measure of genetically effective dispersal (migration) than does *F*_ST_ [64]. The number of migrants between populations, *Nm*, was calculated from ^*S*^*H*_*UA*_. To estimate divergence times using Goldstein *et al*.’s [65] absolute dating method, we first calculated their measure of genetic distance, (δμ)^2^ [65, 66], using Arlequin. The number of generations, τ_gen_, since coalescence, was calculated using the following equation (Eq 2 in [67]):

$$\tau_{\text{gen}}=\frac{{\left(\delta \mu \right)}^{2}}{2\mu }$$

where μ is the microsatellite mutation rate. We used a mutation rate of 10^−3^ for microsatellites in vertebrates [68–70] and a generation time of 10 years [35] to estimate divergence times. If we use a mutation rate an order of magnitude lower (10^−4^) our estimated divergence times are an order of magnitude longer, in tens of thousands rather than thousands of years. In any case, 10^−3^ is an average of a set of values with high variance; the actual rates of the particular loci are what matters and these are unknown. As these divergence estimates are based on only the four loci common to all samples, stochasticity will be high and so their relative values, which will not change if we change mutation rates, are more valid than their absolute values. We provide a range of times (95% confidence intervals), rather than a point estimate [71]. If this confidence interval included 0, the divergence time was not significant. We also supply divergence time estimates based on eight (set A) or nine loci (set B).

Network analysis was implemented in EDENetworks [72] to construct a minimum-spanning tree (MST) based on the genetic distance between populations. An MST is the minimal network necessary to connect all population genetic samples taken at sites in a whole data set. For this purpose, the program plots all sites (nodes) in a network graph with connections (edges) between all nodes. Each edge was weighted according to its pairwise genetic distance ((δμ)^2^). The MST selects a subset of edges that connects all nodes while minimizing the overall genetic distance. The layout of the MST was recalculated 1000 times to generate a 50% bootstrap tree. The resulting network was then manually manipulated (without changing degree of connectedness) to better conform to geographic reality.

## Results

### Geographic patterns of variation within populations

#### Mitochondrial DNA

**Fig 2:**
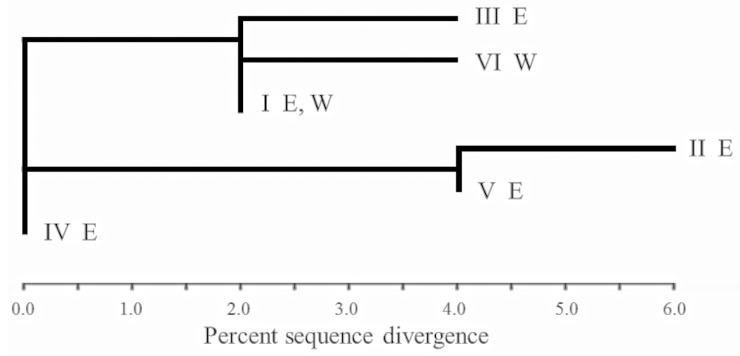
RFLP Haplotypes. Unrooted neighbour-joining tree of six whole mtDNA RFLP haplotypes in *L*. *latifrons* populations. E = east of the Eyrean barrier, W = west of it.

Six RFLP haplotypes were identified across the seven SHN wombat sites surveyed (Table 2 and Fig 2). Five haplotypes (I–V) were found in the 24 eastern (Murraylands) individuals, while the 23 western samples–collected over a much larger geographic area–revealed only haplotypes I and VI. Haplotype I, the only one shared between geographic regions, was also the most common in each region. The average within-site RFLP nucleotide diversity across all seven sampling sites was 2.41% (SE = 0.002, range 0.40–6.63%,Table 2). The average within-site nucleotide diversity for the eastern sites (4.94%) was an order of magnitude higher than that of the western sites (0.52%). All four western sites had significant negative values for Fu’s *F*_*S*_, indicating population expansion or selection (Table 3). Two of the three eastern sites had non-significant *F*_*S*_, but Brookfield had a significant negative *F*_*S*_ (Table 3). Tajima’s *D*, which is less powerful in detecting population expansion or selection than is *F*_*S*_, was significant only for Fowler’s Bay in the west, and even then, only marginally (Table 3).

**Table 2:** Southern blot RFLP compound haplotypes and frequencies, and nucleotide diversity (as percentages) in seven sites of SHN wombats.

		Haplotype						
Site	Region	I	II	III	IV	V	VI	Diversity
Fowler’s Bay	west	3	0	0	0	0	3	0.55
Nundroo	west	2	0	0	0	0	1	0.62
Coorabie	west	4	0	0	0	0	2	0.49
Mount Wedge	west	2	0	0	0	0	6	0.40
**Average (west)**								**0.52**
Swan Reach	east	4	4	0	0	2	0	4.19
Brookfield	east	2	1	1	1	0	0	6.63
Sturt Highway	east	5	2	0	1	1	0	3.99
**Average (east)**								**4.94**

**Table 3:** Fu’s *F*_*S*_ and Tajima’s *D* values, based on RFLP haplotypes, for seven sites of SHN wombats.

Site	Region	*F*_*S*_	*P*	*D*	*P*
Fowler’s Bay	west	−5.95	<0.00001	1.75	0.05
Nundroo	west	−6.35	<0.00001	1.03	0.19
Coorabie	west	−6.35	<0.00001	1.03	0.20
Mount Wedge	west	−5.27	<0.00001	0.41	0.34
Swan Reach	east	−0.94	0.26	0.03	0.48
Brookfield	east	−30.08	<0.00001	−0.75	0.32
Sturt Highway	east	−1.35	0.14	−0.26	0.43

Among the 64 individuals screened by SSCP, two cytochrome *b* haplotypes were evident, named F (Genbank HM008258) and G (Genbank HM008259). Sequencing of 12 SSCP phenotype representatives revealed no further sequence diversity than was evident from SSCP phenotype alone and showed that the two haplotypes differ by only two base pairs (0.5% sequence divergence)–both synonymous, third position transitions. The haplotypes associated perfectly with the two whole-mtDNA RFLP haplotypes identified by Taylor [33]. Haplotype F was present in all sites, but haplotype G was present only in the three eastern sites. The six RFLP haplotypes were nested within the two cytochrome *b* haplotypes: individuals with RFLP haplotypes I, III, IV or VI had cytochrome *b* haplotype F, while individuals with the RFLP haplotypes II or V had cytochrome *b* haplotype G. Cytochrome *b* nucleotide diversity was greater for the eastern (0.17–0.26) than the western sites (all 0).

#### Nuclear DNA

At all 24 sites, the four microsatellite loci common to all samples were polymorphic, with an average of five or more alleles per locus, and expected heterozygosities (*H*_E_) were high (average = 0.72) (Table 1). There was little indication of Wahlund effect or technically problematic loci. The Wahlund effect occurs when a population has more homozygous and fewer heterozygous genotypes than would be expected [73]. This effect would be evidence that wombats from different areas had been pooled in the most recent generation, either naturally, or by our sampling. There were only two significant homozygous excess deviations from HWE out of 96 exact tests (Bramfield, locus 54A; and Poochera, 68CA), both west of the Eyrean barrier. Using all available loci in each set increased the number of significant deviations to only eight (7 of 96 exact tests in set A, 1 of 135 in set B), adding sites east of the Eyrean barrier (Brookfield, locus 3AT; Swan Reach, 3AT; and Wauraltee, 16CA, 54CA, 68CA in set A; Kulpara, 68CA in set B; Nundroo, 71CA, 107 was the only significant western site, in set A). Similarly, the few significant LD tests were not patterned with respect to locus pairs or sites. Even when all available loci were examined, the number of private alleles was highest on the Yorke Peninsula, consistent with its many small and isolated populations (S1 Table). Thus there is little evidence of substructure within sites.

Levels of genetic variation at microsatellite loci differed among the 24 sites (Friedman tests for *H*_E_ *P* = 0.003, for *AR*, *P* = 0.002). There was little or no east–west pattern in level of genetic variation: correlations of *H*_*E*_ and *AR* with longitude were small and non-significant (*H*_*E*_: *r* = 0.034; *AR*: *r* = 0.009; *P* > 0.10). However, there were consistent collections of regional sites that had high or low diversity as identified by the approach specified in Methods (S2 Table). The FWC, with its large population sizes, had relatively high diversity: Nundroo and Coorabie in particular had many significantly high *H*_E_ comparisons with other sites, and these sites plus Ceduna similarly showed high *AR*. Equally, the large Murraylands populations in the east generally had high *H*_E_: Sturt Highway, Swan Reach and Brookfield had many significantly high pairwise comparisons with other sites, and the first two of these sites also showed high *AR*. In contrast, the remaining sites, central to the species range, showed generally low variation with the notable exception of Hiltaba, which had high *H*_E_ and *AR*. Point Pearce and Wallaroo (Yorke Peninsula), and Mount Wedge (Eyre Peninsula), had significantly low *H*_E_ compared to most other locations, and disproportionately low values of *AR* were seen at Point Pearce, Wallaroo and the other Yorke Peninsula site Junkyard, also Mount Wedge, and two Gawler Ranges sites (Scrubby Peak and Rose Swamp).

### Regional population genetic structure

#### Common variation in mitochondrial DNA shared across the Eyrean barrier

There was modest divergence between locations east and west of the Eyrean barrier, based mainly on a few closely related haplotypes being recorded only in the east or only in the west. A hierarchical AMOVA on these mtDNA data indicated east vs. west structuring: between the two regions Φ_CT_ = 0.34, *P* < 0.028; among sites within regions Φ_SC_ = 0, *P* > 0.41; within sites Φ_ST_ = 0.33, *P* < 0.001. It should be noted that the absolute estimates of mtDNA divergence are upwardly biased because we screened known polymorphic RFLP haplotypes, so relative divergences are most relevant. The smallest and the largest haplotype frequency divergences (found by REAP) were between haplotypes in the east (Murraylands) (0.82% similarity between haplotypes II and V; 17.95% between II and III). The two haplotypes seen in the west (I and VI) had low divergence (0.92% similarity).

Cytochrome *b* haplotype F was present at all sites, but haplotype G was detected only in eastern sites. Based on their 0.5% divergence, and assuming 2% divergence per million years [74, 75], these two haplotypes may have diverged approximately 250 000 years ago. Since this derives from only two base pairs, the estimate is highly uncertain, but is consistent with the whole-mtDNA RFLP estimates in being small. This mtDNA divergence pre-dates by many thousands of years the divergence revealed by the microsatellite data (see below).

#### Close relationships in nuclear DNA between locations neighbouring each side of the Eyrean barrier

**Fig 3:**
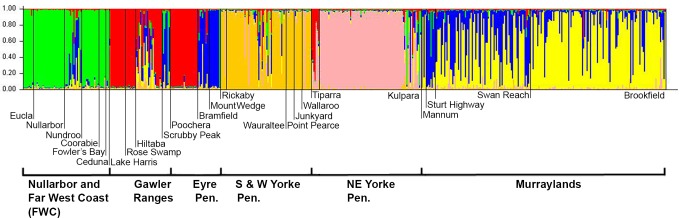
Microsatellite Clusters. Proportion of each of six microsatellite structure clusters represented in individuals from each sampling site, using the four loci in the combined dataset (sets A and B). Columns represent data from individual SHN wombats, divided into sampling sites by vertical narrow dark lines, and organized into geographic regions.

structure identified six microsatellite clusters, which were highly associated with geographic regions: cluster 1 represented individuals mainly from Nullarbor + FWC, cluster 2 Gawler Ranges, cluster 3 Eyre Peninsula, cluster 4 south and west Yorke Peninsula, cluster 5 northeast Yorke Peninsula, and cluster 6 much of the Murraylands (Fig 3, S3 Table). Probability of membership of a single cluster was very clear, 0.9 or greater, for 10 of the 24 sites (S3 Table). There were two sites (Tiparra, Sturt Highway) with the greatest affiliation to clusters outside their geographic area, but in both cases they were also strongly affiliated with their expected cluster. Two other sites, Mannum and Swan Reach, were most strongly associated with their geographic area but were almost equally associated with clusters outside their geographic area. Clusters 1 and 6, the extreme west and east clusters, were also evident using set A alone (eight loci) or set B alone (nine loci). These within-dataset analyses are reported in more detail below.

As would be expected, the six geographic groupings that are also largely reflected in microsatellite cluster membership (S3 Table) were significantly genetically differentiated according to hierarchical amovas of microsatellite data (Table 4). However, the allele frequency variance within these groupings was higher than the variance among, reflecting the complexity of local differentiation. *G* tests of ^*S*^*H*_*UA*_ (measure of gene flow) were significant for only three of 276 pairwise comparisons between sites, indicating restricted gene flow: Hiltaba (Gawler Ranges) vs. three south and west Yorke Peninsula sites—Junkyard (^*S*^*H*_*UA*_ = 0.743, *P* = 0.019), Point Pearce (^*S*^*H*_*UA*_ = 0.728, *P* = 0.019) and Rickaby (^*S*^*H*_*UA*_ = 0.699, *P* = 0.042). Levels of subdivision within geographic groupings varied: average estimates for the Murraylands were relatively low (gene flow high), being ^*S*^*H*_*UA*_ = 0.119, *Nm* = 1.719, and were higher (gene flow lower) for other regions (Nullarbor+FWC, 0.280, 0.310; locations on the Yorke Peninsula 0.342, 0.208; Eyre Peninsula 0.340, 0.210; Gawler Ranges 0.307, 0.258).

**Table 4:** Hierarchical analysis of molecular variance (amova) based on microsatellite data for 24 SHN wombat sample sites, (a) among the six geographic groups identified by structure, and (b) among two groups (East v. West) identified by forcing *K* = 2.

Source of variation	Variance	% total	Probability^a^	*F*-statistics
(a) 6-cluster solution				
Among groups	0.10	10.89	<0.0001	0.109
Among sites within groups	0.04	4.11	<0.0001	0.046
Within sites	0.77	85.00	<0.0001	0.150
(b) 2-cluster solution (East v. West)				
Among groups	0.03	3.79	<0.0001	0.038
Among sites within groups	0.14	15.76	<0.0001	0.164
Within sites	0.71	80.45	<0.0001	0.196

a. Probability of having a significantly higher variance component and *F*-statistic than the observed values by chance alone (1023 permutations).

Divergence times estimated from (δμ)^2^, calculated from four loci common to all samples, showed patterns that were not clearly related to geographic proximity, and showed several relatively close relationships between locations on different sides of the Eyrean barrier (Table 5). These microsatellite data divergence times were in hundreds to thousands of years, rather than the hundreds of thousands of years indicated by the mtDNA data (see above). Between the six clusters, only six divergence times were significant. Nullarbor + FWC showed significant divergence from the Murraylands, south and west Yorke Peninsula, Eyre Peninsula and Gawler Ranges. There was also significant divergence between Eyre Peninsula and south and west Yorke Peninsula, and between south and west Yorke Peninsula and the Murraylands. Recent habitat fragmentation and consequent lower-than-average *H*_*E*_ on the south and west Yorke Peninsula is likely to have inflated the differentiation of these sites (and estimated divergence times) from the nearby Murraylands. Again, this same pattern of results was found using sets A and B alone (as reported in more detail below). The longest significant divergence time in both sets was associated with the extreme west cluster.

**Table 5:** Table of (δμ)^2^ distances between 24 collection sites, calculated by Arlequin from microsatellite data (lower diagonal) and divergence times in years, calculated from these distances (upper diagonal). **Bold** indicates sites within the same cluster identified by STRUCTURE.

Sites	1	2	3	4	5	6	7	8	9	11	10	12	13	14	15	16	17	18	19	20	21	22	23	24
1. Eucla	—	**163**	**309**	**457**	**518**	**2037**	2099	2061	1376	1431	3644	2228	4230	2734	3138	6640	3640	4146	1324	1088	4923	9140	2626	4366
2. Nullarbor	**0.03**	—	**616**	**595**	**782**	**2263**	3100	2892	1550	1742	3779	3079	4995	3859	4434	8399	5034	5635	2150	1922	6115	10049	3361	4999
3. Nundroo	**0.06**	**0.12**	—	**649**	**266**	**1650**	2816	2651	1123	1268	3343	1067	2843	3294	3549	7615	4167	4275	539	982	3374	6938	1491	2878
4. Coorabie	**0.09**	**0.12**	**0.13**	—	**307**	**650**	3178	3050	1930	2217	5246	2663	5059	4554	4915	8701	5304	5969	1384	2380	5762	11020	3860	5710
5. Fowler’s By	**0.10**	**0.16**	**0.05**	**0.06**	—	**845**	2961	2545	908	1156	3508	1274	2906	4072	4336	8404	4776	5793	830	1852	3543	7744	2192	3469
6. Ceduna	**0.41**	**0.45**	**0.33**	**0.13**	**0.17**	—	5649	5346	3181	3752	7431	3106	5700	7588	7795	12275	8170	8667	1761	4414	6286	12009	5060	6747
7. Lake Harris	0.42	0.62	0.56	0.64	0.59	1.13	—	**226**	**2849**	**2212**	4232	3488	4950	407	508	1580	425	2622	3180	1373	4667	9834	3857	5786
8. Rose Swamp	0.41	0.58	0.53	0.61	0.51	1.07	**0.05**	—	**1836**	**1301**	2832	3007	3769	858	1027	2555	943	4007	3323	1734	3809	8374	3326	4731
9. Hiltaba	0.28	0.31	0.22	0.39	0.18	0.64	**0.57**	**0.37**	—	**71**	900	1318	1380	3648	4004	8053	4415	7055	2310	2289	2341	4895	1466	1852
10. Scrubby Pk	0.29	0.35	0.25	0.44	0.23	0.75	**0.44**	**0.26**	**0.01**	—	738	1319	1330	2843	3157	6781	3506	6252	2375	1928	2121	4718	1348	1805
11. Poochera	0.73	0.76	0.67	1.05	0.70	1.49	0.85	0.57	0.18	0.15	—	**2793**	**1502**	4345	4771	8788	5264	8825	5061	3727	2672	3321	1930	1566
12. Bramfield	0.45	0.62	0.21	0.53	0.25	0.62	0.70	0.60	0.26	0.26	**0.56**	—	**765**	3705	3614	7648	4139	4888	707	1556	739	3362	397	1029
13. Mt Wedge	0.85	1.00	0.57	1.01	0.58	1.14	0.99	0.75	0.28	0.27	**0.30**	**0.15**	—	5094	5049	9396	5548	7897	2910	3382	296	1403	678	323
14. Rickaby	0.55	0.77	0.66	0.91	0.81	1.52	0.08	0.17	0.73	0.57	0.87	0.74	1.02	—	**51**	**1064**	**156**	**1497**	3559	994	4622	8817	3420	5333
15. Wauraltee	0.63	0.89	0.71	0.98	0.87	1.56	0.10	0.21	0.80	0.63	0.95	0.72	1.01	**0.01**	—	**879**	**72**	**1299**	3495	1066	4354	8654	3422	5354
16. Pt Pearce	1.33	1.68	1.52	1.74	1.68	2.46	0.32	0.51	1.61	1.36	1.76	1.53	1.88	**0.21**	**0.18**	—	**575**	**2372**	7220	3719	8080	13715	7587	10120
17. Junkyard	0.73	1.01	0.83	1.06	0.96	1.63	0.08	0.19	0.88	0.70	1.05	0.83	1.11	**0.03**	**0.01**	**0.11**	—	**1688**	4031	1566	4745	9499	4131	6124
18. Wallaroo	0.83	1.13	0.86	1.19	1.16	1.73	0.52	0.80	1.41	1.25	1.76	0.98	1.58	**0.30**	**0.26**	**0.47**	**0.34**	—	3455	1377	6696	11389	4706	7501
19. Kulpara	0.26	0.43	0.11	0.28	0.17	0.35	0.64	0.66	0.46	0.47	1.01	0.14	0.58	0.71	0.70	1.44	0.81	0.69	—	**1088**	2720	6859	1548	3141
20. Tiparra	0.22	0.38	0.20	0.48	0.37	0.88	0.27	0.35	0.46	0.39	0.75	0.31	0.68	0.20	0.21	0.74	0.31	0.28	**0.22**	—	3206	6747	1438	3186
21. Sturt Hwy	0.98	1.22	0.67	1.15	0.71	1.26	0.93	0.76	0.47	0.42	0.53	0.15	0.06	0.92	0.87	1.62	0.95	1.34	0.54	0.64	—	**1624**	**833**	**743**
22. Mannum	1.83	2.01	1.39	2.20	1.55	2.40	1.97	1.67	0.98	0.94	0.66	0.67	0.28	1.76	1.73	2.74	1.90	2.28	1.37	1.35	**0.32**	—	**2167**	**891**
23. Swan Rch	0.53	0.67	0.30	0.77	0.44	1.01	0.77	0.67	0.29	0.27	0.39	0.08	0.14	0.68	0.68	1.52	0.83	0.94	0.31	0.29	**0.17**	**0.43**	—	**375**
24. Brookield	0.87	1.00	0.58	1.14	0.69	1.35	1.16	0.95	0.37	0.36	0.31	0.21	0.06	1.07	1.07	2.02	1.22	1.50	0.63	0.64	**0.15**	**0.18**	**0.08**	—

**Fig 4:**
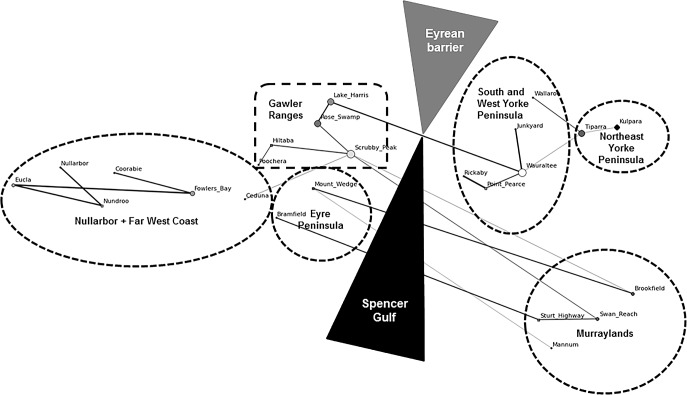
Network Analysis. Minimum-spanning tree based on pairwise genetic distances [(δμ)^2^] arranged approximately by geographic layout of sampling locations. Size of nodes and edges are scaled to the degree of ‘connectedness’ to other populations, lighter colours of edges indicate decreasing connectedness. Dashed lines enclose structure microsatellite clusters.

Network analysis, which allows visualisation of these population relationships, showed numerous connections in genetic similarity between clusters on either side of the Eyrean barrier (Fig 4). Indeed, if populations east and west of the barrier were distinct, we would expect the barrier to break no edges on Fig 4, whereas it breaks six. Consistent with this, forcing structure to identify two clusters yields two groups that are genetically different from each other (Table 4, ^*S*^*H*_*UA*_ = 0.372, amova *P* < 0.0001), but in which the cluster containing eastern sites also included some sites west of the Eyrean barrier (Hiltaba, Bramfield and Mt Wedge). As a result of this cross-gulf relatedness, an *a priori* east–west split separating sites on either side of the Eyrean barrier did not produce significantly different groups (amova *P* = 0.141).

#### Using more loci available within subsets of data

The main findings of the range-wide four-locus analyses were supported by separate analyses using all eight loci available in set A and nine in set B. Because each set sampled different sites, structure found only four clusters, not six, in each set. In both sets, the Murraylands sites east of the Eyrean barrier formed one cluster, and the Nullarbor + FWC and some Eyre Peninsula sites to the west of the barrier formed another cluster. In both sets, the two other clusters were on the Yorke Peninsula, although in set A the northeast Yorke cluster (east of the Eyrean barrier) also included sites from the Eyre Peninsula (west). Hierarchical amova analyses confirmed significant differences in variation among the four clusters identified in each set (set A *P* = 0.032, 2.23% of total variation; set B *P* < 0.0001, 5.71% of variation).

A similar pattern of relative divergence times between sites was seen in estimates based on the combined four-locus data, and estimates using all available loci in sets A and B separately. All divergence times were significant with the exception of one time in set B (between the western—Nullarbor/FWC/Eyre Peninsula—cluster and the south and west Yorke Peninsula cluster). Most importantly, in both data sets, the western cluster was associated with the longest divergence times. The divergence time between the western cluster and the eastern Murraylands cluster was 2.3 times longer than the shortest divergence time. In set A, the longest divergence time (between the western and northeast Yorke Peninsula clusters) ranged from 356,537 to 658,445 years whereas the shortest time, between the Murraylands cluster and the south-and-west Yorke Peninsula cluster, ranged from 19,687 to 177,885 years. In set B, the longest divergence time (between the western and Murraylands clusters) ranged from 16,642 to 807,729 years whereas the shortest time, between the two Yorke Peninsula clusters, ranged from 27,275 to 327,805 years. The different estimates from the different numbers of loci reflects sensitivity of (δμ)^2^ to the number and diversity of loci employed [65, 66]

## Discussion

Our results suggest that geological events may have structured genetic variation in the SHN wombat, but in such a way that neither the Eyrean barrier nor the Spencer Gulf marks a clear genetic break in the centre of the SHN wombat distribution. We found some evidence for genetic differentiation between the most easterly and westerly sampled locations of SHN wombat. This may reflect past influence of the closing of the land bridge, where Spencer Gulf now lies, when the sea level rose after the period of maximum Pleistocene aridity, approximately 17,000 years ago (Fig 1) [14]. The period of maximum aridity also produced the Eyrean barrier north of Spencer Gulf land bridge [25, 26] and the Nullarbor barrier at the western end of the SHN wombat’s current distribution [9]. Molecular data have shown that these two barriers were responsible for major phylogeographic breaks in birds, other vertebrates and plants [9, 27, 28]. We tried to sample SHN wombats 175 km west of Eucla (Fig 1) but the populations there appeared extinct [19]. However, future research using mtDNA from both sides of the Nullarbor barrier may potentially reveal a SHN wombat Pleistocene refuge west of this barrier. For some marsupials, this period of maximum aridity did not present barriers but instead opened a land bridge across Spencer Gulf and to Kangaroo Island [2]. Closing of this land bridge after the Pleistocene arid period may have contributed to changes in genetic diversity in the SHN wombat and speciation in other marsupials.

We examined four scenarios for the potential consequences of the closing of the Spencer Gulf land bridge on patterns of genetic diversity in and among SHN wombat populations. *Scenario 1* proposed that the Eyrean barrier did not prevent gene flow and hence there would be low divergence between populations on either side of the Gulf. The genetic analyses present considerable support for this scenario. First, nuclear variation indicated relatively close genetic similarity of many pairs of locations on opposite sides of the Eyrean barrier / Spencer Gulf, providing evidence for gene flow across the barrier. In particular, the Eyre Peninsula (west) cluster is weakly diverged from the Murraylands (east). The Gawler Ranges (west) also has some strong connections with eastern sites, including in the Murraylands (Fig 4). The geographic arrangement of these east–west connections suggests complex patterns of gene flow across the barrier. While the absolute estimates of divergence times here are coarse [76–78], they are in thousands rather than millions of years, and many east–west site pairs are only as diverged as ones within the same regional cluster. Such patterns are consistent with gene flow occurring during late Pleistocene and Holocene environmental fluctuations, fitting plausible biogeographic scenarios such as movement across the Spencer Gulf land bridge during periods of Pleistocene aridity.

Second, the data are also consistent with only a partial closure of the Spencer Gulf land bridge 10 000 years ago, so that some gene flow was maintained to the north of Spencer Gulf. This partial closure version of *Scenario 1* would account for the moderate diversity in the Eyre Peninsula contact zone compared to the east and west extremes, the only moderate differentiation between east and west, and the weak evidence for expansion from the east. We note that currently, the range of the SHN wombat has become patchy due to European settlement (Fig 1), cutting off gene flow via the narrow band of suitable habitat north of Spencer Gulf.

*Scenario 2* proposed post-land bridge admixture between refuges west and east of Spencer Gulf, and therefore greater diversity in the contact zone(s) between the two refuges. There was also support for this scenario, as two potential refuges were indicated by mtDNA and nuclear DNA variation. While mtDNA divergence was slight, and the most common haplotype shared between east and west, the east had three haplotypes not found in the west and the west had one not found in the east, resulting in a significant AMOVA differentiation test. Nuclear variation was less clearly split, as expected given its higher effective population size but nonetheless there were some relatively large and significant differences between the most westerly and the most easterly sampled locations. The mtDNA cytochrome *b* divergence dates back to approximately 250 000 years ago, well before the Last Glacial Maximum (24 500 BCE), whereas the nuclear DNA divergences are much more recent, less than 100 000 years ago (the longest divergence time in Table 5 is 13 715 years). While these patterns could coincide with refuges caused by mid-Pleistocene aridity, as suggested for other fauna in the region [2], rather than post-Pleistocene refuges, both could reflect isolation by distance, prior to and after the disappearance of the Spencer Gulf land bridge.

Notwithstanding apparent east–west differentiation, the prediction of *Scenario 2* of highest diversity at the point of contact between two refuges was not fulfilled by the data. Average expected heterozygosity (*H*_*E*_) was perhaps even relatively low where eastern and western putative populations would be expected to meet (Table 1). This may reflect recent population reduction and habitat fragmentation effects.

The competing *Scenario 3* –little or no contact between refuges on either side of Spencer Gulf–is inconsistent with the above evidence for low differentiation and inferred gene flow among regions. *Scenario 4* proposed that the species expanded from a single Pleistocene refuge. On the contrary, we found the above evidence for western and eastern differentiation. Fu’s *F*_*S*_ was significant, indicating expansion, for sites from the west and the east, but these results were not replicated using Tajima’s *D*. Although diversity indices tended to be lower in the west compared to the east (Tables 1 and 2), the correlations between longitude and *H*_*E*_ and *AR* were small and not significant, providing no isolation-by-distance evidence for expansion from a single refuge. Thus the data do not support *Scenario 4*.

Overall the data give the greatest support to *Scenario 1* (low divergence between populations on either side of the Spencer Gulf land bridge), plausibly consistent with little impact of the Gulf as a post-Pleistocene barrier. Nevertheless, across the species’ broad range, some geographic groups might have been separated for some thousands of years.

### Conservation Implications

Microsatellite and mtDNA data are consistent with SHN wombat population differentiation caused by periods of Pleistocene aridity, as has been established for many other taxa in the region [2, 9, 27, 28]. Even if we assume a microsatellite mutation rate an order of magnitude faster than the vertebrate average (10^−2^ instead of 10^−3^), divergence between the east and west ends of the species’ distribution seems to predate the 500 years that is conventionally accepted as the time when humans began to impact on natural evolution [79]. Thus, any proposal for translocation to enhance gene flow between these two extremes would warrant risk assessment [79, 80], even though there are no ecological differences between the regions where SHN wombats are currently found (including these east and west extremes) [40]. But additional information would be valuable before any determination of appropriate conservation interventions, including more robust estimates of divergence times and gene flow based on more loci and ideally conducted by simulation-based coalescent analysis, and even more importantly, relative fitness consequences of inbreeding and outbreeding depression [79, 81]. The effects of translocations between substantially distant populations should be observed first in fenced-off experimental plots before repeating the procedure in the field. Regardless, in the case of extremely small, isolated populations that are suffering inbreeding depression or likely to do so, making them prone to extinction, gene flow from nearby but distinct populations is likely to be sufficiently beneficial to outweigh the competing risks of delaying action [23]. We do not advocate expensive risk assessment, but a cautious balanced approach is necessary for managers to take appropriate conservation actions, weighing up costs and benefits and the urgency of the situation. For some smaller populations sampled over a decade ago, intervention may already be too late, as they are likely extinct.

Six distinct geographic population groupings were identified. The largest population of SHN wombats exists towards the western edge of its distribution, the FWC, on which basis this would be the most likely population to survive into the future. However, this population represents only one of six genotypic groups, and only the western (much less variable) mitochondrial grouping that putatively reflects only one of two mid-Pleistocene refuges. Much genetic information and evolutionary potential could be lost if genetic variation from the other five genotypic zones were not also conserved.

The Yorke Peninsula populations have diverged from the others at least in part as a consequence of fragmentation and genetic drift. However, two sites, Tiparra and Wauraltee, preserve private alleles, and may harbour genetic variation of value to the species as a whole. Only seven animals were found with thorough sampling at the Tiparra site.

In summary, we found evidence that the SHN wombat species is genetically subdivided into six genotypic clusters, some of which may have persisted for thousands of years. There is some evidence for two refuges, or possibly isolation by distance, on each side of the land bridge across the Spencer Gulf that existed during periods of Pleistocene aridity. This substructure should be considered in efforts to preserve the maximum potential for adaptation in the species, including conservation interventions involving facilitated gene flow [23]. It also presents a basis for thorough investigation of the genome-wide structure of the species given the availability of current efficient genomic screening [82]. Future studies that combine new genetic sampling in the contact zone west of Spencer Gulf and additional markers such as 50 000 single nucleotide polymorphisms in a spatial analysis with contemporary environmental data (e.g., land use change, geology, hydrology and vegetation types) would provide substantial insights into the combined influence of past climatic changes and current land use on SHN wombat population viability. This approach would support the identification and prioritization of locations for amelioration of land degradation and facilitation of natural gene flow.

## Supporting Information

S1 File MS-Excel raw data. **Microsatellite genotypes for 24 *L*. *latifrons* populations.** Set A: 12 populations, 8 loci. Set B: 16 populations 9 loci.
(XLSX)

S2 File Arlequin project file. **Mitochondrial DNA haplotypes for 7 *L*. *latifrons* populations.** Frequencies of 6 RFLP haplotypes.
(ARP)

S1 Table Allele Frequencies. Allele frequencies in samples from all collection sites analysed for *Lasiorhinus latifrons*.
(DOCX)

S2 Table Comparisons among All Pairs of Sites. Significance of Wilcoxon paired-samples signed-ranks tests of genetic diversity measures tested between all pairs of 24 sampling sites of SHN wombats.
(DOCX)

S3 Table Cluster Membership. Average probability of membership for individuals in six genetic clusters inferred by structure, using microsatellite data (four loci) from 24 *L*. *latifrons* populations.
(DOCX)

S1 Text Supplementary Methods. Chelex Extractions of Hair Samples. Phenol/IAC Extractions of Hair, Blood, and Tissue Samples. Amplification of Microsatellite Loci. Genotyping Process.
(DOCX)
