# Supplementary material for: Evidence of Subdivisions on Evolutionary Timescales in a Large, Declining Marsupial Distributed across a Phylogeographic Barrier
Source: PLoS One. 2016 Oct 12;11(10):e0162789. doi: 10.1371/journal.pone.0162789 (PMC5061365; doi:10.1371/journal.pone.0162789)
Supplement: S1 Table — Allele frequencies in samples from all collection sites analysed for Lasiorhinus latifrons. (DOCX) [file pone.0162789.s003.docx]

**S1 Table. Allele Frequencies.** Allele frequencies in samples from all collection sites analysed for *Lasiorhinus latifrons*. Population numbers coincide with Table 1 of the article. Private alleles found in one site are marked in bold.

|  | Eucla | Null-arbor | Nun-droo | Coo-rabie | Fowl.Bay | Ced-una | Lake Harris | Rose Swamp | Hil-taba | Poo-chera | Scrubby Peak | Bram-field | Mount Wedge | Rick-aby | Wau-raltee | Point Pearce | Junkyard | Wal-laroo | Tipa-rra | Kul-para | Sturt Hwy | Man-num | Swan Reach | Brook-field |
| --- | --- | --- | --- | --- | --- | --- | --- | --- | --- | --- | --- | --- | --- | --- | --- | --- | --- | --- | --- | --- | --- | --- | --- | --- |
| **Lla54CA** | | | | | | | | | | | | | | | | | | | | | | | | |
| 1 | 0.000 | 0.000 | 0.000 | 0.000 | 0.083 | 0.167 | 0.000 | 0.000 | 0.000 | 0.000 | 0.000 | 0.000 | 0.000 | 0.000 | 0.000 | 0.000 | 0.000 | 0.000 | 0.000 | 0.000 | 0.000 | 0.000 | 0.000 | 0.000 |
| 2 | 0.000 | 0.000 | 0.000 | 0.000 | 0.000 | 0.000 | 0.000 | 0.000 | 0.000 | 0.000 | 0.000 | 0.000 | 0.000 | 0.000 | 0.000 | 0.000 | 0.000 | 0.063 | 0.214 | 0.435 | 0.000 | 0.000 | 0.067 | 0.013 |
| 3 | 0.389 | 0.556 | 0.056 | 0.300 | 0.167 | 0.167 | 0.000 | 0.000 | 0.000 | 0.000 | 0.000 | 0.000 | 0.000 | 0.000 | 0.056 | 0.000 | 0.000 | 0.063 | 0.000 | 0.000 | 0.000 | 0.000 | 0.000 | 0.000 |
| 4 | 0.000 | 0.000 | 0.111 | 0.100 | 0.000 | 0.000 | 0.000 | 0.000 | 0.000 | 0.000 | 0.000 | 0.000 | 0.050 | 0.000 | 0.014 | 0.000 | 0.000 | 0.000 | 0.000 | 0.000 | 0.000 | 0.000 | 0.000 | 0.000 |
| 5 | 0.000 | 0.000 | **0.111** | 0.000 | 0.000 | 0.000 | 0.000 | 0.000 | 0.000 | 0.000 | 0.000 | 0.000 | 0.000 | 0.000 | 0.000 | 0.000 | 0.000 | 0.000 | 0.000 | 0.000 | 0.000 | 0.000 | 0.000 | 0.000 |
| 6 | 0.444 | 0.370 | 0.500 | 0.450 | 0.333 | 0.333 | 0.000 | 0.000 | 0.000 | 0.167 | 0.000 | 0.000 | 0.050 | 0.200 | 0.097 | 0.417 | 0.500 | 0.625 | 0.143 | 0.143 | 0.125 | 0.125 | 0.299 | 0.254 |
| 7 | 0.000 | 0.000 | 0.000 | 0.000 | 0.000 | 0.167 | 0.000 | 0.000 | 0.000 | 0.000 | 0.000 | 0.500 | 0.000 | 0.000 | 0.014 | 0.000 | 0.000 | 0.000 | 0.000 | 0.000 | 0.000 | 0.000 | 0.000 | 0.000 |
| 8 | 0.000 | 0.000 | 0.056 | 0.100 | 0.000 | 0.000 | 0.000 | 0.000 | 0.000 | 0.000 | 0.000 | 0.000 | 0.000 | 0.000 | 0.014 | 0.000 | 0.000 | 0.000 | 0.071 | 0.084 | 0.125 | 0.000 | 0.171 | 0.110 |
| 9 | 0.000 | 0.000 | 0.000 | 0.000 | 0.000 | 0.000 | 0.000 | 0.000 | 0.000 | 0.000 | 0.000 | 0.000 | 0.000 | 0.200 | 0.222 | 0.000 | 0.000 | 0.188 | 0.000 | 0.000 | 0.000 | 0.000 | 0.000 | 0.004 |
| 10 | 0.056 | 0.000 | 0.000 | 0.000 | 0.000 | 0.000 | 0.167 | 0.111 | 0.250 | 0.000 | 0.146 | 0.000 | 0.000 | 0.000 | 0.028 | 0.000 | 0.000 | 0.000 | 0.143 | 0.000 | 0.000 | 0.000 | 0.000 | 0.000 |
| 11 | 0.000 | 0.000 | 0.000 | 0.000 | 0.000 | 0.000 | 0.792 | 0.500 | 0.375 | 0.000 | 0.083 | 0.000 | 0.000 | 0.000 | 0.000 | 0.000 | 0.000 | 0.000 | 0.071 | 0.000 | 0.000 | 0.000 | 0.000 | 0.000 |
| 12 | 0.000 | 0.000 | 0.000 | 0.000 | 0.000 | 0.000 | 0.000 | 0.111 | 0.250 | 0.000 | 0.625 | 0.000 | 0.000 | 0.000 | 0.042 | 0.000 | 0.000 | 0.000 | 0.357 | 0.071 | 0.000 | 0.000 | 0.079 | 0.089 |
| 13 | 0.000 | 0.019 | 0.111 | 0.000 | 0.250 | 0.000 | 0.000 | 0.000 | 0.000 | 0.083 | 0.000 | 0.000 | 0.000 | 0.400 | 0.319 | 0.167 | 0.000 | 0.000 | 0.000 | 0.000 | 0.000 | 0.000 | 0.000 | 0.000 |
| 14 | 0.000 | 0.000 | 0.000 | 0.000 | 0.167 | 0.167 | 0.000 | 0.000 | 0.000 | 0.000 | 0.000 | 0.000 | 0.000 | 0.100 | 0.014 | 0.000 | 0.071 | 0.000 | 0.000 | 0.130 | 0.000 | 0.000 | 0.000 | 0.000 |
| 15 | 0.111 | 0.037 | 0.000 | 0.000 | 0.000 | 0.000 | 0.000 | 0.000 | 0.000 | 0.000 | 0.000 | 0.000 | 0.000 | 0.000 | 0.000 | 0.000 | 0.000 | 0.000 | 0.000 | 0.000 | 0.000 | 0.000 | 0.000 | 0.000 |
| 16 | 0.000 | 0.019 | 0.056 | 0.050 | 0.000 | 0.000 | 0.000 | 0.000 | 0.000 | 0.500 | 0.000 | 0.250 | 0.550 | 0.000 | 0.000 | 0.000 | 0.000 | 0.000 | 0.000 | 0.000 | 0.000 | 0.000 | 0.000 | 0.000 |
| 17 | 0.000 | 0.000 | 0.000 | 0.000 | 0.000 | 0.000 | 0.000 | 0.000 | 0.000 | 0.000 | 0.000 | 0.167 | 0.100 | 0.000 | 0.000 | 0.000 | 0.000 | 0.000 | 0.000 | 0.000 | 0.000 | 0.000 | 0.049 | 0.106 |
| 18 | 0.000 | 0.000 | 0.000 | 0.000 | 0.000 | 0.000 | 0.000 | 0.000 | 0.000 | 0.000 | 0.000 | 0.000 | 0.000 | 0.100 | 0.028 | 0.000 | 0.071 | 0.000 | 0.000 | 0.019 | 0.313 | 0.375 | 0.098 | 0.034 |
| 19 | 0.000 | 0.000 | 0.000 | 0.000 | 0.000 | 0.000 | 0.042 | 0.278 | 0.125 | 0.000 | 0.146 | 0.083 | 0.000 | 0.000 | 0.014 | 0.000 | 0.000 | 0.000 | 0.000 | 0.000 | 0.000 | 0.000 | 0.000 | 0.008 |
| 20 | 0.000 | 0.000 | 0.000 | 0.000 | 0.000 | 0.000 | 0.000 | 0.000 | 0.000 | 0.250 | 0.000 | 0.000 | 0.250 | 0.000 | 0.014 | 0.000 | 0.000 | 0.000 | 0.000 | 0.117 | 0.125 | 0.000 | 0.006 | 0.055 |
| 21 | 0.000 | 0.000 | 0.000 | 0.000 | 0.000 | 0.000 | 0.000 | 0.000 | 0.000 | 0.000 | 0.000 | 0.000 | 0.000 | 0.000 | 0.125 | 0.417 | 0.357 | 0.063 | 0.000 | 0.000 | 0.000 | 0.000 | 0.006 | 0.000 |
| 22 | 0.000 | 0.000 | 0.000 | 0.000 | 0.000 | 0.000 | 0.000 | 0.000 | 0.000 | 0.000 | 0.000 | 0.000 | 0.000 | 0.000 | 0.000 | 0.000 | 0.000 | 0.000 | 0.000 | 0.000 | 0.313 | 0.500 | 0.226 | 0.326 |
| **Lla67CA** | | | | | | | | | | | | | | | | | | | | | | | | |
| 1 | 0.000 | 0.000 | 0.000 | 0.000 | 0.000 | 0.000 | 0.000 | 0.000 | 0.000 | 0.000 | 0.000 | 0.000 | 0.000 | 0.400 | 0.311 | 0.667 | 0.571 | 0.250 | 0.000 | 0.000 | 0.000 | 0.000 | 0.000 | 0.000 |
| 2 | 0.000 | 0.000 | 0.000 | 0.000 | 0.000 | 0.000 | 0.000 | 0.000 | 0.000 | 0.000 | 0.000 | 0.000 | 0.000 | 0.000 | **0.022** | 0.000 | 0.000 | 0.000 | 0.000 | 0.000 | 0.000 | 0.000 | 0.000 | 0.000 |
| 3 | 0.000 | 0.000 | 0.000 | 0.000 | 0.000 | 0.000 | 0.000 | 0.000 | 0.000 | 0.000 | 0.000 | 0.000 | 0.000 | 0.200 | 0.300 | 0.083 | 0.071 | 0.375 | 0.000 | 0.000 | 0.000 | 0.000 | 0.000 | 0.000 |
| 4 | 0.000 | 0.000 | 0.000 | 0.000 | 0.000 | 0.000 | 0.000 | 0.000 | 0.000 | 0.000 | 0.000 | 0.000 | 0.000 | 0.000 | 0.000 | 0.000 | 0.000 | 0.000 | **0.083** | 0.000 | 0.000 | 0.000 | 0.000 | 0.000 |
| 5 | 0.000 | 0.000 | 0.000 | 0.000 | 0.000 | 0.000 | 0.923 | 0.667 | 0.026 | 0.000 | 0.000 | 0.000 | 0.000 | 0.000 | 0.000 | 0.000 | 0.000 | 0.000 | 0.417 | 0.006 | 0.000 | 0.000 | 0.000 | 0.000 |
| 6 | 0.056 | 0.000 | 0.036 | 0.000 | 0.000 | 0.000 | 0.000 | 0.000 | 0.000 | 0.000 | 0.000 | 0.000 | 0.000 | 0.000 | 0.000 | 0.000 | 0.000 | 0.000 | 0.000 | 0.000 | 0.000 | 0.000 | 0.000 | 0.000 |
| 7 | 0.111 | 0.000 | 0.000 | 0.033 | 0.000 | 0.000 | 0.000 | 0.000 | 0.000 | 0.071 | 0.000 | 0.000 | 0.000 | 0.000 | 0.000 | 0.250 | 0.000 | 0.000 | 0.000 | 0.083 | 0.250 | 0.000 | 0.012 | 0.000 |
| 8 | 0.333 | 0.130 | 0.143 | 0.267 | 0.000 | 0.000 | 0.000 | 0.000 | 0.000 | 0.000 | 0.000 | 0.056 | 0.000 | 0.100 | 0.067 | 0.000 | 0.000 | 0.250 | 0.000 | 0.179 | 0.063 | 0.167 | 0.151 | 0.124 |
| 9 | 0.167 | 0.148 | 0.429 | 0.100 | 0.167 | 0.167 | 0.000 | 0.222 | 0.368 | 0.357 | 0.083 | 0.389 | 0.250 | 0.000 | 0.011 | 0.000 | 0.000 | 0.000 | 0.083 | 0.095 | 0.063 | 0.000 | 0.307 | 0.208 |
| 10 | 0.000 | 0.704 | 0.071 | 0.367 | 0.583 | 0.167 | 0.000 | 0.056 | 0.237 | 0.143 | 0.792 | 0.056 | 0.000 | 0.000 | 0.033 | 0.000 | 0.000 | 0.063 | 0.167 | 0.458 | 0.188 | 0.000 | 0.048 | 0.000 |
| 11 | 0.278 | 0.019 | 0.071 | 0.067 | 0.000 | 0.167 | 0.077 | 0.056 | 0.158 | 0.071 | 0.104 | 0.000 | 0.000 | 0.000 | 0.089 | 0.000 | 0.071 | 0.000 | 0.250 | 0.000 | 0.000 | 0.000 | 0.151 | 0.133 |
| 12 | 0.056 | 0.000 | 0.000 | 0.000 | 0.000 | 0.000 | 0.000 | 0.000 | 0.000 | 0.000 | 0.021 | 0.278 | 0.400 | 0.300 | 0.167 | 0.000 | 0.286 | 0.063 | 0.000 | 0.083 | 0.000 | 0.000 | 0.090 | 0.058 |
| 13 | 0.000 | 0.000 | 0.107 | 0.167 | 0.250 | 0.500 | 0.000 | 0.000 | 0.000 | 0.214 | 0.000 | 0.056 | 0.100 | 0.000 | 0.000 | 0.000 | 0.000 | 0.000 | 0.000 | 0.000 | 0.000 | 0.000 | 0.000 | 0.004 |
| 14 | 0.000 | 0.000 | 0.071 | 0.000 | 0.000 | 0.000 | 0.000 | 0.000 | 0.000 | 0.143 | 0.000 | 0.167 | 0.250 | 0.000 | 0.000 | 0.000 | 0.000 | 0.000 | 0.000 | 0.006 | 0.313 | 0.833 | 0.133 | 0.088 |
| 15 | 0.000 | 0.000 | 0.071 | 0.000 | 0.000 | 0.000 | 0.000 | 0.000 | 0.132 | 0.000 | 0.000 | 0.000 | 0.000 | 0.000 | 0.000 | 0.000 | 0.000 | 0.000 | 0.000 | 0.089 | 0.125 | 0.000 | 0.108 | 0.385 |
| 16 | 0.000 | 0.000 | 0.000 | 0.000 | 0.000 | 0.000 | 0.000 | 0.000 | **0.079** | 0.000 | 0.000 | 0.000 | 0.000 | 0.000 | 0.000 | 0.000 | 0.000 | 0.000 | 0.000 | 0.000 | 0.000 | 0.000 | 0.000 | 0.000 |
| **Lla68CA** | | | | | | | | | | | | | | | | | | | | | | | | |
| 1 | 0.000 | 0.000 | 0.000 | 0.000 | 0.000 | 0.000 | 0.000 | 0.000 | 0.000 | 0.000 | 0.000 | 0.000 | 0.000 | 0.000 | 0.029 | 0.071 | 0.000 | 0.313 | 0.143 | 0.176 | 0.000 | 0.000 | 0.000 | 0.000 |
| 2 | 0.000 | 0.000 | 0.000 | 0.000 | 0.000 | 0.000 | 0.000 | 0.000 | 0.050 | 0.000 | 0.000 | 0.000 | 0.000 | 0.000 | 0.020 | 0.000 | 0.000 | 0.000 | 0.143 | 0.017 | 0.250 | 0.125 | 0.169 | 0.286 |
| 3 | 0.000 | 0.074 | 0.000 | 0.000 | 0.000 | 0.000 | 0.000 | 0.000 | 0.000 | 0.000 | 0.000 | 0.000 | 0.000 | 0.000 | 0.000 | 0.000 | 0.000 | 0.000 | 0.000 | 0.205 | 0.063 | 0.250 | 0.042 | 0.000 |
| 4 | 0.056 | 0.000 | 0.000 | 0.143 | 0.083 | 0.167 | 0.000 | 0.000 | 0.000 | 0.000 | 0.000 | 0.050 | 0.000 | 0.100 | 0.039 | 0.000 | 0.214 | 0.000 | 0.000 | 0.011 | 0.000 | 0.000 | 0.006 | 0.000 |
| 5 | 0.444 | 0.222 | 0.167 | 0.286 | 0.250 | 0.333 | 0.036 | 0.000 | 0.000 | 0.000 | 0.000 | 0.150 | 0.000 | 0.000 | 0.000 | 0.000 | 0.000 | 0.000 | 0.000 | 0.011 | 0.000 | 0.125 | 0.060 | 0.017 |
| 6 | 0.000 | 0.000 | 0.400 | 0.000 | 0.000 | 0.000 | 0.214 | 0.222 | 0.300 | 0.357 | 0.396 | 0.700 | 0.350 | 0.000 | 0.049 | 0.000 | 0.000 | 0.000 | 0.000 | 0.028 | 0.438 | 0.375 | 0.337 | 0.188 |
| 7 | 0.000 | 0.000 | 0.200 | 0.321 | 0.250 | 0.167 | 0.214 | 0.111 | 0.100 | 0.143 | 0.063 | 0.100 | 0.600 | 0.000 | 0.441 | 0.214 | 0.000 | 0.438 | 0.071 | 0.051 | 0.188 | 0.000 | 0.199 | 0.158 |
| 8 | 0.000 | 0.000 | 0.000 | 0.000 | 0.000 | 0.000 | 0.036 | 0.000 | 0.000 | 0.000 | 0.000 | 0.000 | 0.000 | 0.700 | 0.314 | 0.714 | 0.643 | 0.188 | 0.000 | 0.057 | 0.000 | 0.000 | 0.000 | 0.000 |
| 9 | 0.000 | 0.000 | 0.000 | 0.000 | 0.000 | 0.000 | 0.000 | 0.000 | 0.000 | 0.000 | 0.000 | 0.000 | 0.000 | 0.200 | 0.078 | 0.000 | 0.143 | 0.000 | 0.143 | 0.210 | 0.000 | 0.000 | 0.084 | 0.154 |
| 10 | 0.000 | 0.000 | 0.000 | 0.000 | 0.000 | 0.000 | 0.000 | 0.000 | 0.100 | 0.000 | 0.000 | 0.000 | 0.000 | 0.000 | 0.010 | 0.000 | 0.000 | 0.063 | 0.500 | 0.216 | 0.000 | 0.000 | 0.006 | 0.000 |
| 11 | 0.000 | 0.000 | 0.000 | 0.000 | 0.000 | 0.000 | 0.393 | 0.389 | 0.175 | 0.071 | 0.354 | 0.000 | 0.000 | 0.000 | 0.000 | 0.000 | 0.000 | 0.000 | 0.000 | 0.006 | 0.063 | 0.125 | 0.090 | 0.175 |
| 12 | 0.056 | 0.315 | 0.067 | 0.036 | 0.083 | 0.167 | 0.107 | 0.278 | 0.025 | 0.000 | 0.000 | 0.000 | 0.050 | 0.000 | 0.000 | 0.000 | 0.000 | 0.000 | 0.000 | 0.000 | 0.000 | 0.000 | 0.000 | 0.000 |
| 13 | 0.278 | 0.259 | 0.067 | 0.071 | 0.333 | 0.000 | 0.000 | 0.000 | 0.000 | 0.000 | 0.000 | 0.000 | 0.000 | 0.000 | 0.010 | 0.000 | 0.000 | 0.000 | 0.000 | 0.011 | 0.000 | 0.000 | 0.000 | 0.000 |
| 14 | 0.000 | 0.000 | 0.000 | **0.036** | 0.000 | 0.000 | 0.000 | 0.000 | 0.000 | 0.000 | 0.000 | 0.000 | 0.000 | 0.000 | 0.000 | 0.000 | 0.000 | 0.000 | 0.000 | 0.000 | 0.000 | 0.000 | 0.000 | 0.000 |
| 15 | 0.056 | 0.130 | 0.000 | 0.000 | 0.000 | 0.000 | 0.000 | 0.000 | 0.000 | 0.000 | 0.000 | 0.000 | 0.000 | 0.000 | 0.000 | 0.000 | 0.000 | 0.000 | 0.000 | 0.000 | 0.000 | 0.000 | 0.000 | 0.000 |
| 16 | 0.111 | 0.000 | 0.100 | 0.107 | 0.000 | 0.167 | 0.000 | 0.000 | 0.025 | 0.429 | 0.000 | 0.000 | 0.000 | 0.000 | 0.010 | 0.000 | 0.000 | 0.000 | 0.000 | 0.000 | 0.000 | 0.000 | 0.006 | 0.017 |
| 17 | 0.000 | 0.000 | 0.000 | 0.000 | 0.000 | 0.000 | 0.000 | 0.000 | 0.225 | 0.000 | 0.188 | 0.000 | 0.000 | 0.000 | 0.000 | 0.000 | 0.000 | 0.000 | 0.000 | 0.000 | 0.000 | 0.000 | 0.000 | 0.000 |
| 18 | 0.000 | 0.000 | 0.000 | 0.000 | 0.000 | 0.000 | 0.000 | 0.000 | 0.000 | 0.000 | 0.000 | 0.000 | 0.000 | 0.000 | 0.000 | 0.000 | 0.000 | 0.000 | 0.000 | 0.000 | 0.000 | 0.000 | 0.000 | **0.004** |
| **Lla71CA** | | | | | | | | | | | | | | | | | | | | | | | | |
| 1 | 0.278 | 0.185 | 0.045 | 0.233 | 0.167 | 0.500 | 0.154 | 0.250 | 0.071 | 0.000 | 0.104 | 0.083 | 0.000 | 0.000 | 0.012 | 0.000 | 0.000 | 0.000 | 0.000 | 0.303 | 0.000 | 0.000 | 0.000 | 0.000 |
| 2 | 0.000 | 0.019 | 0.182 | 0.233 | 0.250 | 0.167 | 0.000 | 0.000 | 0.000 | 0.000 | 0.000 | 0.000 | 0.000 | 0.000 | 0.000 | 0.000 | 0.000 | 0.000 | 0.000 | 0.000 | 0.000 | 0.000 | 0.000 | 0.000 |
| 3 | 0.000 | 0.000 | 0.000 | 0.000 | 0.000 | 0.000 | 0.000 | 0.000 | 0.036 | 0.000 | 0.000 | 0.000 | 0.000 | 0.000 | 0.000 | 0.000 | 0.000 | 0.000 | 0.000 | 0.000 | 0.000 | 0.000 | 0.024 | 0.000 |
| 4 | 0.000 | 0.000 | 0.000 | 0.000 | 0.000 | 0.000 | 0.000 | 0.000 | 0.000 | 0.000 | 0.000 | 0.000 | 0.000 | 0.000 | 0.000 | 0.000 | 0.000 | 0.000 | 0.000 | 0.000 | 0.000 | 0.000 | **0.018** | 0.000 |
| 5 | 0.000 | 0.000 | 0.091 | 0.000 | 0.000 | 0.000 | 0.000 | 0.000 | 0.000 | 0.000 | 0.000 | 0.083 | 0.000 | 0.100 | 0.107 | 0.250 | 0.333 | 0.063 | 0.000 | 0.000 | 0.000 | 0.000 | 0.000 | 0.000 |
| 6 | 0.000 | 0.167 | 0.091 | 0.067 | 0.083 | 0.000 | 0.231 | 0.063 | 0.000 | 0.000 | 0.188 | 0.000 | 0.000 | 0.000 | 0.012 | 0.000 | 0.000 | 0.000 | 0.000 | 0.026 | 0.375 | 0.000 | 0.024 | 0.000 |
| 7 | 0.000 | 0.000 | 0.000 | 0.000 | 0.000 | 0.000 | 0.000 | 0.000 | **0.036** | 0.000 | 0.000 | 0.000 | 0.000 | 0.000 | 0.000 | 0.000 | 0.000 | 0.000 | 0.000 | 0.000 | 0.000 | 0.000 | 0.000 | 0.000 |
| 8 | 0.000 | 0.000 | 0.000 | 0.067 | 0.000 | 0.000 | 0.000 | 0.000 | 0.321 | 0.000 | 0.000 | 0.167 | 0.056 | 0.000 | 0.012 | 0.000 | 0.000 | 0.000 | 0.000 | 0.007 | 0.125 | 0.000 | 0.199 | 0.230 |
| 9 | 0.000 | 0.019 | 0.045 | 0.033 | 0.000 | 0.000 | 0.269 | 0.063 | 0.179 | 0.167 | 0.292 | 0.500 | 0.500 | 0.600 | 0.571 | 0.583 | 0.333 | 0.625 | 0.750 | 0.309 | 0.125 | 0.250 | 0.271 | 0.091 |
| 10 | 0.000 | 0.000 | 0.000 | 0.000 | 0.083 | 0.000 | 0.154 | 0.125 | 0.179 | 0.083 | 0.000 | 0.000 | 0.000 | 0.000 | 0.000 | 0.000 | 0.083 | 0.000 | 0.083 | 0.053 | 0.000 | 0.000 | 0.048 | 0.187 |
| 11 | 0.722 | 0.481 | 0.227 | 0.100 | 0.000 | 0.167 | 0.154 | 0.438 | 0.036 | 0.250 | 0.104 | 0.000 | 0.000 | 0.100 | 0.107 | 0.000 | 0.000 | 0.125 | 0.083 | 0.000 | 0.250 | 0.000 | 0.006 | 0.000 |
| 12 | 0.000 | 0.130 | 0.318 | 0.267 | 0.417 | 0.167 | 0.038 | 0.063 | 0.071 | 0.417 | 0.313 | 0.167 | 0.444 | 0.100 | 0.060 | 0.083 | 0.250 | 0.063 | 0.083 | 0.303 | 0.000 | 0.000 | 0.084 | 0.126 |
| 13 | 0.000 | 0.000 | 0.000 | 0.000 | 0.000 | 0.000 | 0.000 | 0.000 | 0.071 | 0.083 | 0.000 | 0.000 | 0.000 | 0.100 | 0.119 | 0.083 | 0.000 | 0.125 | 0.000 | 0.000 | 0.125 | 0.000 | 0.042 | 0.000 |
| 14 | 0.000 | 0.000 | 0.000 | 0.000 | 0.000 | 0.000 | 0.000 | 0.000 | 0.000 | 0.000 | 0.000 | 0.000 | 0.000 | 0.000 | 0.000 | 0.000 | 0.000 | 0.000 | 0.000 | 0.000 | 0.000 | 0.750 | 0.283 | 0.365 |
| **Lla3AT (Set A)** | | | | | | | | | | | | | | | | | | | | | | | | |
| 1 |  |  | 0.000 | 0.500 | 0.000 | 0.000 |  |  | 0.000 |  |  | 0.000 | 0.000 |  | 0.000 |  |  |  |  | 0.000 | 0.125 |  | 0.037 | 0.000 |
| 2 |  |  | 0.000 | 0.000 | 0.000 | 0.000 |  |  | 0.000 |  |  | 0.000 | 0.000 |  | 0.000 |  |  |  |  | 0.000 | 0.000 |  | **0.012** | 0.000 |
| 3 |  |  | 0.000 | 0.500 | 0.000 | 0.000 |  |  | 0.000 |  |  | 0.000 | 0.000 |  | 0.063 |  |  |  |  | 0.000 | 0.000 |  | 0.025 | 0.000 |
| 4 |  |  | 0.000 | 0.000 | 0.000 | 0.000 |  |  | 0.500 |  |  | 0.000 | 0.000 |  | 0.000 |  |  |  |  | 0.000 | 0.000 |  | 0.031 | 0.000 |
| 5 |  |  | 0.000 | 0.000 | 0.000 | 0.000 |  |  | 0.250 |  |  | 0.000 | 0.000 |  | 0.000 |  |  |  |  | 0.000 | 0.000 |  | 0.006 | 0.000 |
| 6 |  |  | 0.091 | 0.000 | 0.167 | 0.333 |  |  | 0.000 |  |  | 0.000 | 0.063 |  | 0.000 |  |  |  |  | 0.000 | 0.000 |  | 0.000 | 0.000 |
| 7 |  |  | 0.000 | 0.000 | 0.000 | 0.167 |  |  | 0.125 |  |  | 0.000 | 0.000 |  | 0.250 |  |  |  |  | 0.000 | 0.000 |  | 0.025 | 0.000 |
| 8 |  |  | 0.000 | 0.000 | 0.083 | 0.000 |  |  | 0.000 |  |  | 0.000 | 0.000 |  | 0.000 |  |  |  |  | 0.167 | 0.000 |  | 0.216 | 0.000 |
| 9 |  |  | 0.091 | 0.000 | 0.000 | 0.000 |  |  | 0.000 |  |  | 0.000 | 0.000 |  | 0.000 |  |  |  |  | 0.000 | 0.000 |  | 0.173 | 0.167 |
| 10 |  |  | 0.091 | 0.000 | 0.000 | 0.000 |  |  | 0.000 |  |  | 0.000 | 0.000 |  | 0.000 |  |  |  |  | 0.000 | 0.000 |  | 0.062 | 0.133 |
| 11 |  |  | 0.091 | 0.000 | 0.083 | 0.167 |  |  | 0.000 |  |  | 0.000 | 0.000 |  | 0.000 |  |  |  |  | 0.000 | 0.000 |  | 0.000 | 0.000 |
| 12 |  |  | 0.000 | 0.000 | 0.167 | 0.000 |  |  | 0.000 |  |  | 0.000 | 0.000 |  | 0.188 |  |  |  |  | 0.167 | 0.000 |  | 0.019 | 0.000 |
| 13 |  |  | 0.045 | 0.000 | 0.000 | 0.000 |  |  | 0.000 |  |  | 0.000 | 0.625 |  | 0.188 |  |  |  |  | 0.000 | 0.563 |  | 0.191 | 0.100 |
| 14 |  |  | 0.045 | 0.000 | 0.250 | 0.000 |  |  | 0.125 |  |  | 0.500 | 0.125 |  | 0.000 |  |  |  |  | 0.417 | 0.000 |  | 0.025 | 0.100 |
| 15 |  |  | 0.136 | 0.000 | 0.000 | 0.000 |  |  | 0.000 |  |  | 0.000 | 0.063 |  | 0.000 |  |  |  |  | 0.000 | 0.000 |  | 0.000 | 0.000 |
| 16 |  |  | 0.227 | 0.000 | 0.167 | 0.000 |  |  | 0.000 |  |  | 0.000 | 0.000 |  | 0.188 |  |  |  |  | 0.250 | 0.000 |  | 0.006 | 0.000 |
| 17 |  |  | 0.000 | 0.000 | 0.083 | 0.333 |  |  | 0.000 |  |  | 0.000 | 0.000 |  | 0.063 |  |  |  |  | 0.000 | 0.000 |  | 0.019 | 0.033 |
| 18 |  |  | 0.182 | 0.000 | 0.000 | 0.000 |  |  | 0.000 |  |  | 0.500 | 0.000 |  | 0.000 |  |  |  |  | 0.000 | 0.313 |  | 0.105 | 0.300 |
| 19 |  |  | 0.000 | 0.000 | 0.000 | 0.000 |  |  | 0.000 |  |  | 0.000 | 0.125 |  | 0.000 |  |  |  |  | 0.000 | 0.000 |  | 0.043 | 0.167 |
| 20 |  |  | 0.000 | 0.000 | 0.000 | 0.000 |  |  | 0.000 |  |  | 0.000 | 0.000 |  | 0.000 |  |  |  |  | 0.000 | 0.000 |  | **0.006** | 0.000 |
| 21 |  |  | 0.000 | 0.000 | 0.000 | 0.000 |  |  | 0.000 |  |  | 0.000 | 0.000 |  | **0.063** |  |  |  |  | 0.000 | 0.000 |  | 0.000 | 0.000 |
| **Lla16CA (Set A)** | | | | | | | | | | | | | | | | | | | | | | | | |
| 1 |  |  | 0.000 | 0.000 | 0.000 | 0.000 |  |  | **0.033** |  |  | 0.000 | 0.000 |  | 0.000 |  |  |  |  | 0.000 | 0.000 |  | 0.000 | 0.000 |
| 2 |  |  | 0.100 | 0.000 | 0.000 | 0.333 |  |  | 0.000 |  |  | 0.000 | 0.050 |  | 0.000 |  |  |  |  | 0.179 | 0.000 |  | 0.000 | 0.000 |
| 3 |  |  | 0.067 | 0.050 | 0.000 | 0.000 |  |  | 0.033 |  |  | 0.000 | 0.000 |  | 0.068 |  |  |  |  | 0.143 | 0.250 |  | 0.177 | 0.156 |
| 4 |  |  | 0.000 | 0.100 | 0.000 | 0.000 |  |  | 0.067 |  |  | 0.000 | 0.000 |  | 0.273 |  |  |  |  | 0.000 | 0.000 |  | 0.000 | 0.000 |
| 5 |  |  | 0.000 | 0.100 | 0.000 | 0.333 |  |  | 0.000 |  |  | 0.000 | 0.000 |  | 0.000 |  |  |  |  | 0.000 | 0.000 |  | 0.000 | 0.000 |
| 6 |  |  | 0.300 | 0.550 | 0.250 | 0.333 |  |  | 0.267 |  |  | 0.250 | 0.100 |  | 0.114 |  |  |  |  | 0.357 | 0.438 |  | 0.390 | 0.469 |
| 7 |  |  | 0.533 | 0.200 | 0.750 | 0.000 |  |  | 0.433 |  |  | 0.750 | 0.850 |  | 0.386 |  |  |  |  | 0.286 | 0.313 |  | 0.433 | 0.375 |
| 8 |  |  | 0.000 | 0.000 | 0.000 | 0.000 |  |  | 0.133 |  |  | 0.000 | 0.000 |  | 0.000 |  |  |  |  | 0.036 | 0.000 |  | 0.000 | 0.000 |
| 9 |  |  | 0.000 | 0.000 | 0.000 | 0.000 |  |  | 0.033 |  |  | 0.000 | 0.000 |  | 0.068 |  |  |  |  | 0.000 | 0.000 |  | 0.000 | 0.000 |
| 10 |  |  | 0.000 | 0.000 | 0.000 | 0.000 |  |  | 0.000 |  |  | 0.000 | 0.000 |  | **0.045** |  |  |  |  | 0.000 | 0.000 |  | 0.000 | 0.000 |
| 11 |  |  | 0.000 | 0.000 | 0.000 | 0.000 |  |  | 0.000 |  |  | 0.000 | 0.000 |  | **0.045** |  |  |  |  | 0.000 | 0.000 |  | 0.000 | 0.000 |
| **Lla55A (Set A)** | | | | | | | | | | | | | | | | | | | | | | | | |
| 1 |  |  | 0.000 | 0.000 | 0.000 | 0.000 |  |  | 0.000 |  |  | 0.000 | 0.000 |  | 0.000 |  |  |  |  | **0.071** | 0.000 |  | 0.000 | 0.000 |
| 2 |  |  | 0.000 | 0.000 | 0.000 | 0.000 |  |  | 0.071 |  |  | 0.000 | 0.000 |  | 0.000 |  |  |  |  | 0.071 | 0.000 |  | 0.000 | 0.000 |
| 3 |  |  | 0.286 | 0.286 | 0.000 | 0.500 |  |  | 0.571 |  |  | 0.278 | 0.050 |  | 0.022 |  |  |  |  | 0.214 | 0.563 |  | 0.439 | 0.750 |
| 4 |  |  | 0.429 | 0.179 | 0.417 | 0.167 |  |  | 0.048 |  |  | 0.056 | 0.650 |  | 0.261 |  |  |  |  | 0.393 | 0.188 |  | 0.159 | 0.063 |
| 5 |  |  | 0.286 | 0.536 | 0.583 | 0.333 |  |  | 0.310 |  |  | 0.667 | 0.300 |  | 0.717 |  |  |  |  | 0.250 | 0.250 |  | 0.402 | 0.188 |
| **Lkr107 (Set A)** | | | | | | | | | | | | | | | | | | | | | | | | |
| 1 |  |  | 0.000 | 0.000 | 0.000 | 0.000 |  |  | **0.667** |  |  | 0.000 | 0.000 |  | 0.000 |  |  |  |  | 0.000 | 0.000 |  | 0.000 | 0.000 |
| 2 |  |  | 0.000 | 0.000 | 0.000 | 0.000 |  |  | 0.000 |  |  | 0.000 | 0.150 |  | 0.000 |  |  |  |  | 0.050 | 0.000 |  | 0.000 | 0.000 |
| 3 |  |  | 0.000 | 0.000 | 0.000 | 0.000 |  |  | 0.000 |  |  | 0.143 | 0.000 |  | 0.000 |  |  |  |  | 0.000 | 0.000 |  | 0.006 | 0.031 |
| 4 |  |  | 0.000 | 0.500 | 0.167 | 0.000 |  |  | 0.000 |  |  | 0.000 | 0.050 |  | 0.038 |  |  |  |  | 0.100 | 0.000 |  | 0.000 | 0.000 |
| 5 |  |  | 0.400 | 0.000 | 0.083 | 0.167 |  |  | 0.000 |  |  | 0.000 | 0.000 |  | 0.000 |  |  |  |  | 0.100 | 0.000 |  | 0.000 | 0.000 |
| 6 |  |  | 0.100 | 0.000 | 0.000 | 0.000 |  |  | 0.000 |  |  | 0.000 | 0.000 |  | 0.000 |  |  |  |  | 0.100 | 0.000 |  | 0.000 | 0.000 |
| 7 |  |  | 0.000 | 0.000 | 0.000 | 0.000 |  |  | 0.000 |  |  | 0.000 | 0.000 |  | 0.000 |  |  |  |  | 0.050 | 0.000 |  | 0.024 | 0.000 |
| 8 |  |  | 0.000 | 0.500 | 0.000 | 0.000 |  |  | 0.000 |  |  | 0.000 | 0.000 |  | 0.000 |  |  |  |  | 0.000 | 0.000 |  | 0.104 | 0.031 |
| 9 |  |  | 0.050 | 0.000 | 0.000 | 0.000 |  |  | 0.000 |  |  | 0.000 | 0.000 |  | 0.000 |  |  |  |  | 0.000 | 0.000 |  | 0.061 | 0.031 |
| 10 |  |  | 0.000 | 0.000 | 0.000 | 0.000 |  |  | 0.083 |  |  | 0.000 | 0.000 |  | 0.269 |  |  |  |  | 0.000 | 0.063 |  | 0.262 | 0.406 |
| 11 |  |  | 0.000 | 0.000 | 0.000 | 0.000 |  |  | 0.250 |  |  | 0.000 | 0.000 |  | 0.000 |  |  |  |  | 0.050 | 0.000 |  | 0.024 | 0.000 |
| 12 |  |  | 0.000 | 0.000 | 0.000 | 0.000 |  |  | 0.000 |  |  | 0.714 | 0.600 |  | 0.115 |  |  |  |  | 0.000 | 0.625 |  | 0.396 | 0.281 |
| 13 |  |  | 0.000 | 0.000 | 0.000 | 0.000 |  |  | 0.000 |  |  | 0.000 | **0.050** |  | 0.000 |  |  |  |  | 0.000 | 0.000 |  | 0.000 | 0.000 |
| 14 |  |  | 0.100 | 0.000 | 0.000 | 0.000 |  |  | 0.000 |  |  | 0.000 | 0.000 |  | 0.231 |  |  |  |  | 0.050 | 0.250 |  | 0.061 | 0.188 |
| 15 |  |  | 0.000 | 0.000 | 0.000 | 0.000 |  |  | 0.000 |  |  | 0.000 | 0.000 |  | **0.231** |  |  |  |  | 0.000 | 0.000 |  | 0.000 | 0.000 |
| 16 |  |  | 0.050 | 0.000 | 0.000 | 0.333 |  |  | 0.000 |  |  | 0.000 | 0.050 |  | 0.000 |  |  |  |  | 0.150 | 0.000 |  | 0.000 | 0.000 |
| 17 |  |  | 0.050 | 0.000 | 0.000 | 0.000 |  |  | 0.000 |  |  | 0.000 | 0.000 |  | 0.077 |  |  |  |  | 0.000 | 0.000 |  | 0.000 | 0.000 |
| 18 |  |  | 0.000 | 0.000 | 0.167 | 0.000 |  |  | 0.000 |  |  | 0.000 | 0.000 |  | 0.038 |  |  |  |  | 0.000 | 0.000 |  | 0.000 | 0.000 |
| 19 |  |  | **0.200** | 0.000 | 0.000 | 0.000 |  |  | 0.000 |  |  | 0.000 | 0.000 |  | 0.000 |  |  |  |  | 0.000 | 0.000 |  | 0.000 | 0.000 |
| 20 |  |  | 0.000 | 0.000 | 0.333 | 0.333 |  |  | 0.000 |  |  | 0.000 | 0.000 |  | 0.000 |  |  |  |  | 0.100 | 0.000 |  | 0.000 | 0.000 |
| 21 |  |  | 0.000 | 0.000 | 0.000 | 0.000 |  |  | 0.000 |  |  | 0.000 | 0.000 |  | 0.000 |  |  |  |  | **0.150** | 0.000 |  | 0.000 | 0.000 |
| 22 |  |  | 0.000 | 0.000 | 0.000 | 0.000 |  |  | 0.000 |  |  | 0.000 | 0.100 |  | 0.000 |  |  |  |  | 0.000 | 0.000 |  | 0.006 | 0.031 |
| 23 |  |  | 0.050 | 0.000 | 0.167 | 0.000 |  |  | 0.000 |  |  | 0.000 | 0.000 |  | 0.000 |  |  |  |  | 0.000 | 0.000 |  | 0.000 | 0.000 |
| 24 |  |  | 0.000 | 0.000 | 0.000 | 0.167 |  |  | 0.000 |  |  | 0.143 | 0.000 |  | 0.000 |  |  |  |  | 0.000 | 0.000 |  | 0.000 | 0.000 |
| 25 |  |  | 0.000 | 0.000 | 0.083 | 0.000 |  |  | 0.000 |  |  | 0.000 | 0.000 |  | 0.000 |  |  |  |  | 0.100 | 0.063 |  | 0.055 | 0.000 |
| **Lk13 (Set B)** | | | | | | | | | | | | | | | | | | | | | | | | |
| 1 | 0.000 | 0.000 |  |  |  |  | 0.000 | 0.000 |  | 0.000 | 0.000 |  |  | 0.000 | **0.100** | 0.000 | 0.000 | 0.000 | 0.000 | 0.000 |  | 0.000 |  | 0.000 |
| 2 | 0.000 | 0.000 |  |  |  |  | 0.000 | 0.000 |  | 0.375 | 0.000 |  |  | 0.500 | 0.400 | 0.800 | 0.333 | 0.438 | 0.900 | 0.000 |  | 0.000 |  | 0.000 |
| 3 | 0.000 | 0.000 |  |  |  |  | 0.000 | 0.000 |  | 0.000 | 0.000 |  |  | 0.000 | 0.000 | 0.000 | 0.000 | 0.000 | 0.000 | 0.702 |  | 0.000 |  | 0.495 |
| 4 | **0.375** | 0.000 |  |  |  |  | 0.000 | 0.000 |  | 0.000 | 0.000 |  |  | 0.000 | 0.000 | 0.000 | 0.000 | 0.000 | 0.000 | 0.000 |  | 0.000 |  | 0.000 |
| 5 | 0.000 | **0.643** |  |  |  |  | 0.000 | 0.000 |  | 0.000 | 0.000 |  |  | 0.000 | 0.000 | 0.000 | 0.000 | 0.000 | 0.000 | 0.000 |  | 0.000 |  | 0.000 |
| 6 | 0.000 | 0.000 |  |  |  |  | 0.250 | 0.188 |  | 0.000 | 0.000 |  |  | 0.250 | 0.233 | 0.100 | 0.000 | 0.188 | 0.100 | 0.000 |  | 0.250 |  | 0.000 |
| 7 | 0.000 | 0.000 |  |  |  |  | 0.000 | 0.000 |  | 0.000 | 0.000 |  |  | 0.000 | 0.000 | 0.000 | 0.000 | 0.000 | 0.000 | 0.219 |  | 0.000 |  | 0.066 |
| 8 | 0.500 | 0.000 |  |  |  |  | 0.000 | 0.125 |  | 0.000 | 0.406 |  |  | 0.250 | 0.267 | 0.100 | 0.667 | 0.375 | 0.000 | 0.000 |  | 0.250 |  | 0.000 |
| 9 | 0.000 | 0.190 |  |  |  |  | 0.000 | 0.000 |  | 0.000 | 0.000 |  |  | 0.000 | 0.000 | 0.000 | 0.000 | 0.000 | 0.000 | 0.000 |  | 0.000 |  | 0.297 |
| 10 | 0.000 | **0.024** |  |  |  |  | 0.000 | 0.000 |  | 0.000 | 0.000 |  |  | 0.000 | 0.000 | 0.000 | 0.000 | 0.000 | 0.000 | 0.000 |  | 0.000 |  | 0.000 |
| 11 | 0.000 | 0.000 |  |  |  |  | 0.000 | 0.000 |  | 0.000 | 0.000 |  |  | 0.000 | 0.000 | 0.000 | 0.000 | 0.000 | 0.000 | 0.000 |  | 0.000 |  | **0.005** |
| 12 | 0.000 | 0.000 |  |  |  |  | 0.000 | 0.000 |  | 0.000 | 0.000 |  |  | 0.000 | 0.000 | 0.000 | 0.000 | 0.000 | 0.000 | 0.000 |  | 0.000 |  | **0.027** |
| 13 | 0.000 | 0.000 |  |  |  |  | 0.000 | 0.000 |  | 0.000 | 0.281 |  |  | 0.000 | 0.000 | 0.000 | 0.000 | 0.000 | 0.000 | 0.000 |  | 0.500 |  | 0.000 |
| 14 | 0.000 | 0.000 |  |  |  |  | 0.000 | 0.000 |  | 0.000 | 0.000 |  |  | 0.000 | 0.000 | 0.000 | 0.000 | 0.000 | 0.000 | 0.000 |  | 0.000 |  | **0.110** |
| 15 | 0.125 | 0.000 |  |  |  |  | 0.750 | 0.688 |  | 0.500 | 0.313 |  |  | 0.000 | 0.000 | 0.000 | 0.000 | 0.000 | 0.000 | 0.000 |  | 0.000 |  | 0.000 |
| 16 | 0.000 | 0.000 |  |  |  |  | 0.000 | 0.000 |  | 0.125 | 0.000 |  |  | 0.000 | 0.000 | 0.000 | 0.000 | 0.000 | 0.000 | 0.000 |  | 0.000 |  | 0.000 |
| 17 | 0.000 | 0.000 |  |  |  |  | 0.000 | 0.000 |  | 0.000 | 0.000 |  |  | 0.000 | 0.000 | 0.000 | 0.000 | 0.000 | 0.000 | **0.079** |  | 0.000 |  | 0.000 |
| 18 | 0.000 | **0.143** |  |  |  |  | 0.000 | 0.000 |  | 0.000 | 0.000 |  |  | 0.000 | 0.000 | 0.000 | 0.000 | 0.000 | 0.000 | 0.000 |  | 0.000 |  | 0.000 |
| **Lk21 (Set B)** | | | | | | | | | | | | | | | | | | | | | | | | |
| 1 | 0.000 | 0.000 |  |  |  |  | 0.000 | 0.000 |  | 0.000 | 0.042 |  |  | 0.000 | 0.022 | 0.000 | 0.167 | 0.000 | 0.250 | 0.000 |  | 0.000 |  | 0.000 |
| 2 | 0.000 | 0.000 |  |  |  |  | 0.077 | 0.056 |  | 0.000 | 0.271 |  |  | 0.000 | 0.043 | 0.000 | 0.083 | 0.000 | 0.417 | 0.110 |  | 0.000 |  | 0.000 |
| 3 | 0.000 | 0.000 |  |  |  |  | 0.000 | 0.000 |  | 0.071 | 0.000 |  |  | 0.700 | 0.435 | 0.167 | 0.167 | 0.286 | 0.000 | 0.000 |  | 0.125 |  | 0.193 |
| 4 | 0.000 | 0.000 |  |  |  |  | 0.000 | 0.000 |  | 0.000 | 0.000 |  |  | 0.000 | 0.000 | 0.000 | **0.250** | 0.000 | 0.000 | 0.000 |  | 0.000 |  | 0.000 |
| 5 | 0.000 | 0.000 |  |  |  |  | 0.000 | 0.000 |  | 0.000 | 0.000 |  |  | 0.100 | 0.087 | 0.083 | 0.167 | 0.071 | 0.167 | 0.000 |  | 0.000 |  | 0.000 |
| 6 | 0.000 | 0.000 |  |  |  |  | 0.000 | 0.000 |  | 0.000 | 0.000 |  |  | 0.000 | 0.152 | 0.583 | 0.083 | 0.143 | 0.000 | 0.048 |  | 0.000 |  | 0.020 |
| 7 | 0.000 | 0.000 |  |  |  |  | 0.000 | 0.000 |  | 0.000 | 0.021 |  |  | 0.000 | 0.000 | 0.083 | 0.000 | 0.000 | 0.000 | 0.007 |  | 0.250 |  | 0.272 |
| 8 | 0.278 | 0.426 |  |  |  |  | 0.000 | 0.000 |  | 0.000 | 0.000 |  |  | 0.000 | 0.000 | 0.000 | 0.000 | 0.000 | 0.000 | 0.000 |  | 0.500 |  | 0.010 |
| 9 | 0.167 | 0.185 |  |  |  |  | 0.000 | 0.167 |  | 0.071 | 0.104 |  |  | 0.100 | 0.043 | 0.083 | 0.000 | 0.071 | 0.000 | 0.582 |  | 0.000 |  | 0.267 |
| 10 | 0.444 | 0.352 |  |  |  |  | 0.154 | 0.278 |  | 0.214 | 0.292 |  |  | 0.100 | 0.087 | 0.000 | 0.083 | 0.000 | 0.000 | 0.048 |  | 0.000 |  | 0.188 |
| 11 | 0.056 | 0.037 |  |  |  |  | 0.577 | 0.056 |  | 0.143 | 0.000 |  |  | 0.000 | 0.000 | 0.000 | 0.000 | 0.000 | 0.000 | 0.000 |  | 0.000 |  | 0.000 |
| 12 | 0.000 | 0.000 |  |  |  |  | 0.000 | 0.000 |  | 0.071 | 0.125 |  |  | 0.000 | 0.022 | 0.000 | 0.000 | 0.000 | 0.000 | 0.027 |  | 0.000 |  | 0.000 |
| 13 | 0.000 | 0.000 |  |  |  |  | 0.038 | 0.278 |  | 0.357 | 0.146 |  |  | 0.000 | 0.000 | 0.000 | 0.000 | 0.000 | 0.000 | 0.000 |  | 0.000 |  | 0.000 |
| 14 | 0.056 | 0.000 |  |  |  |  | 0.154 | 0.111 |  | 0.000 | 0.000 |  |  | 0.000 | 0.109 | 0.000 | 0.000 | 0.429 | 0.000 | 0.000 |  | 0.000 |  | 0.000 |
| 15 | 0.000 | 0.000 |  |  |  |  | 0.000 | 0.056 |  | 0.000 | 0.000 |  |  | 0.000 | 0.000 | 0.000 | 0.000 | 0.000 | 0.000 | 0.158 |  | 0.000 |  | 0.000 |
| 16 | 0.000 | 0.000 |  |  |  |  | 0.000 | 0.000 |  | 0.071 | 0.000 |  |  | 0.000 | 0.000 | 0.000 | 0.000 | 0.000 | 0.167 | 0.021 |  | 0.125 |  | 0.050 |
| **Lk23 (Set B)** | | | | | | | | | | | | | | | | | | | | | | | | |
| 1 | 0.000 | 0.000 |  |  |  |  | 0.000 | 0.000 |  | 0.000 | 0.000 |  |  | 0.000 | 0.000 | 0.000 | 0.000 | 0.000 | 0.000 | 0.000 |  | 0.000 |  | **0.050** |
| 2 | 0.000 | 0.192 |  |  |  |  | 0.000 | 0.000 |  | 0.357 | 0.000 |  |  | 0.000 | 0.000 | 0.000 | 0.000 | 0.125 | 0.500 | 0.308 |  | 0.000 |  | 0.000 |
| 3 | 0.056 | 0.000 |  |  |  |  | 0.500 | 0.333 |  | 0.571 | 0.021 |  |  | 0.100 | 0.021 | 0.000 | 0.000 | 0.000 | 0.000 | 0.000 |  | 0.000 |  | 0.000 |
| 4 | 0.056 | 0.154 |  |  |  |  | 0.000 | 0.000 |  | 0.000 | 0.000 |  |  | 0.400 | 0.271 | 0.000 | 0.000 | 0.188 | 0.000 | 0.205 |  | 0.000 |  | 0.010 |
| 5 | 0.000 | 0.000 |  |  |  |  | 0.077 | 0.111 |  | 0.000 | 0.521 |  |  | 0.000 | 0.021 | 0.083 | 0.083 | 0.500 | 0.000 | 0.301 |  | 0.000 |  | 0.000 |
| 6 | 0.444 | 0.481 |  |  |  |  | 0.000 | 0.000 |  | 0.000 | 0.000 |  |  | 0.000 | 0.021 | 0.000 | 0.167 | 0.000 | 0.214 | 0.082 |  | 0.500 |  | 0.619 |
| 7 | 0.444 | 0.173 |  |  |  |  | 0.000 | 0.000 |  | 0.000 | 0.000 |  |  | 0.000 | 0.250 | 0.500 | 0.667 | 0.000 | 0.000 | 0.000 |  | 0.000 |  | 0.054 |
| 8 | 0.000 | 0.000 |  |  |  |  | 0.000 | 0.000 |  | 0.071 | 0.083 |  |  | 0.000 | 0.042 | 0.167 | 0.000 | 0.000 | 0.286 | 0.000 |  | 0.250 |  | 0.005 |
| 9 | 0.000 | 0.000 |  |  |  |  | 0.115 | 0.000 |  | 0.000 | 0.000 |  |  | 0.000 | 0.000 | 0.083 | 0.000 | 0.000 | 0.000 | 0.000 |  | 0.000 |  | 0.079 |
| 10 | 0.000 | 0.000 |  |  |  |  | 0.000 | 0.111 |  | 0.000 | 0.271 |  |  | 0.300 | 0.104 | 0.000 | 0.083 | 0.063 | 0.000 | 0.103 |  | 0.000 |  | 0.124 |
| 11 | 0.000 | 0.000 |  |  |  |  | 0.000 | 0.111 |  | 0.000 | 0.104 |  |  | 0.200 | 0.271 | 0.167 | 0.000 | 0.125 | 0.000 | 0.000 |  | 0.000 |  | 0.000 |
| 12 | 0.000 | 0.000 |  |  |  |  | 0.000 | 0.000 |  | 0.000 | 0.000 |  |  | 0.000 | 0.000 | 0.000 | 0.000 | 0.000 | 0.000 | 0.000 |  | 0.000 |  | **0.035** |
| 13 | 0.000 | 0.000 |  |  |  |  | 0.308 | 0.333 |  | 0.000 | 0.000 |  |  | 0.000 | 0.000 | 0.000 | 0.000 | 0.000 | 0.000 | 0.000 |  | 0.125 |  | 0.000 |
| 14 | 0.000 | 0.000 |  |  |  |  | 0.000 | 0.000 |  | 0.000 | 0.000 |  |  | 0.000 | 0.000 | 0.000 | 0.000 | 0.000 | 0.000 | 0.000 |  | 0.125 |  | 0.025 |
| **Lk37 (Set B)** | | | | | | | | | | | | | | | | | | | | | | | | |
| 1 | 0.389 | 0.038 |  |  |  |  | 0.192 | 0.167 |  | 0.417 | 0.271 |  |  | 0.000 | 0.045 | 0.250 | 0.083 | 0.000 | 0.333 | 0.000 |  | 0.000 |  | 0.343 |
| 2 | 0.000 | 0.000 |  |  |  |  | 0.000 | 0.000 |  | 0.167 | 0.000 |  |  | 0.000 | 0.045 | 0.000 | 0.000 | 0.125 | 0.417 | 0.087 |  | 0.000 |  | 0.108 |
| 3 | **0.111** | 0.000 |  |  |  |  | 0.000 | 0.000 |  | 0.000 | 0.000 |  |  | 0.000 | 0.000 | 0.000 | 0.000 | 0.000 | 0.000 | 0.000 |  | 0.000 |  | 0.000 |
| 4 | 0.000 | 0.000 |  |  |  |  | 0.000 | 0.000 |  | 0.000 | 0.000 |  |  | 0.000 | 0.000 | 0.000 | 0.000 | 0.000 | 0.000 | 0.000 |  | 0.000 |  | **0.059** |
| 5 | 0.000 | 0.000 |  |  |  |  | 0.000 | 0.000 |  | 0.000 | 0.000 |  |  | 0.000 | 0.000 | 0.000 | 0.000 | 0.000 | 0.000 | 0.007 |  | 0.333 |  | 0.000 |
| 6 | 0.000 | 0.000 |  |  |  |  | 0.000 | 0.000 |  | 0.000 | 0.000 |  |  | 0.000 | 0.000 | 0.000 | 0.000 | 0.000 | 0.000 | 0.000 |  | 0.333 |  | 0.225 |
| 7 | 0.000 | 0.000 |  |  |  |  | 0.000 | 0.000 |  | 0.000 | 0.000 |  |  | 0.000 | 0.000 | 0.000 | 0.000 | 0.000 | 0.000 | 0.000 |  | 0.167 |  | 0.093 |
| 8 | 0.000 | 0.000 |  |  |  |  | 0.000 | 0.000 |  | 0.000 | 0.000 |  |  | 0.000 | 0.182 | 0.000 | 0.000 | 0.063 | 0.000 | 0.000 |  | 0.000 |  | 0.000 |
| 9 | 0.000 | 0.000 |  |  |  |  | 0.000 | 0.000 |  | 0.000 | 0.000 |  |  | 0.200 | 0.045 | 0.000 | 0.000 | 0.000 | 0.000 | 0.000 |  | 0.000 |  | 0.000 |
| 10 | 0.000 | 0.000 |  |  |  |  | 0.000 | 0.000 |  | 0.000 | 0.000 |  |  | 0.000 | 0.182 | 0.000 | 0.000 | 0.125 | 0.000 | 0.000 |  | 0.000 |  | 0.000 |
| 11 | 0.000 | 0.000 |  |  |  |  | 0.000 | 0.000 |  | 0.000 | 0.000 |  |  | 0.000 | 0.000 | 0.000 | 0.000 | 0.000 | 0.000 | **0.457** |  | 0.000 |  | 0.000 |
| 12 | 0.000 | 0.000 |  |  |  |  | 0.500 | 0.389 |  | 0.000 | 0.000 |  |  | 0.400 | 0.341 | 0.333 | 0.583 | 0.688 | 0.250 | 0.043 |  | 0.167 |  | 0.093 |
| 13 | 0.167 | 0.712 |  |  |  |  | 0.269 | 0.389 |  | 0.083 | 0.271 |  |  | 0.400 | 0.159 | 0.417 | 0.333 | 0.000 | 0.000 | 0.406 |  | 0.000 |  | 0.078 |
| 14 | 0.333 | 0.250 |  |  |  |  | 0.038 | 0.056 |  | 0.333 | 0.458 |  |  | 0.000 | 0.000 | 0.000 | 0.000 | 0.000 | 0.000 | 0.000 |  | 0.000 |  | 0.000 |
| **Ll2 (Set B)** | | | | | | | | | | | | | | | | | | | | | | | | |
| 1 | 0.500 | 0.018 |  |  |  |  | 0.000 | 0.000 |  | 0.143 | 0.000 |  |  | 0.000 | 0.000 | 0.000 | 0.000 | 0.000 | 0.000 | 0.000 |  | 0.000 |  | 0.000 |
| 2 | 0.000 | **0.750** |  |  |  |  | 0.000 | 0.000 |  | 0.000 | 0.000 |  |  | 0.000 | 0.000 | 0.000 | 0.000 | 0.000 | 0.000 | 0.000 |  | 0.000 |  | 0.000 |
| 3 | 0.222 | 0.000 |  |  |  |  | 0.000 | 0.000 |  | 0.071 | 0.000 |  |  | 0.000 | 0.042 | 0.000 | 0.000 | 0.063 | 0.083 | 0.000 |  | 0.500 |  | 0.103 |
| 4 | 0.222 | 0.161 |  |  |  |  | 0.000 | 0.000 |  | 0.000 | 0.000 |  |  | 0.000 | 0.000 | 0.071 | 0.071 | 0.000 | 0.000 | 0.000 |  | 0.000 |  | 0.000 |
| 5 | 0.000 | 0.000 |  |  |  |  | 0.154 | 0.167 |  | 0.357 | 0.000 |  |  | 0.500 | 0.375 | 0.214 | 0.357 | 0.125 | 0.000 | 0.000 |  | 0.250 |  | 0.064 |
| 6 | 0.000 | 0.000 |  |  |  |  | 0.462 | 0.333 |  | 0.071 | 0.500 |  |  | 0.000 | 0.000 | 0.071 | 0.000 | 0.000 | 0.000 | 0.000 |  | 0.000 |  | 0.000 |
| 7 | 0.000 | 0.071 |  |  |  |  | 0.000 | 0.000 |  | 0.143 | 0.000 |  |  | 0.100 | 0.021 | 0.000 | 0.000 | 0.125 | 0.000 | 0.062 |  | 0.000 |  | 0.098 |
| 8 | 0.056 | 0.000 |  |  |  |  | 0.000 | 0.000 |  | 0.214 | 0.000 |  |  | 0.100 | 0.125 | 0.214 | 0.143 | 0.125 | 0.000 | 0.267 |  | 0.000 |  | 0.000 |
| 9 | 0.000 | 0.000 |  |  |  |  | 0.154 | 0.056 |  | 0.000 | 0.271 |  |  | 0.200 | 0.188 | 0.143 | 0.214 | 0.125 | 0.333 | 0.130 |  | 0.000 |  | 0.000 |
| 10 | 0.000 | 0.000 |  |  |  |  | 0.077 | 0.333 |  | 0.000 | 0.146 |  |  | 0.000 | 0.083 | 0.143 | 0.071 | 0.063 | 0.583 | 0.185 |  | 0.000 |  | 0.000 |
| 11 | 0.000 | 0.000 |  |  |  |  | 0.000 | 0.000 |  | 0.000 | **0.083** |  |  | 0.000 | 0.000 | 0.000 | 0.000 | 0.000 | 0.000 | 0.000 |  | 0.000 |  | 0.000 |
| 12 | 0.000 | 0.000 |  |  |  |  | 0.000 | 0.000 |  | 0.000 | 0.000 |  |  | 0.100 | 0.146 | 0.143 | 0.143 | 0.375 | 0.000 | 0.260 |  | 0.000 |  | 0.000 |
| 13 | 0.000 | 0.000 |  |  |  |  | 0.077 | 0.111 |  | 0.000 | 0.000 |  |  | 0.000 | 0.021 | 0.000 | 0.000 | 0.000 | 0.000 | 0.096 |  | 0.125 |  | 0.196 |
| 14 | 0.000 | 0.000 |  |  |  |  | 0.000 | 0.000 |  | 0.000 | 0.000 |  |  | 0.000 | 0.000 | 0.000 | 0.000 | 0.000 | 0.000 | 0.000 |  | 0.125 |  | 0.451 |
| 15 | 0.000 | 0.000 |  |  |  |  | 0.077 | 0.000 |  | 0.000 | 0.000 |  |  | 0.000 | 0.000 | 0.000 | 0.000 | 0.000 | 0.000 | 0.000 |  | 0.000 |  | 0.078 |
| 16 | 0.000 | 0.000 |  |  |  |  | 0.000 | 0.000 |  | 0.000 | 0.000 |  |  | 0.000 | 0.000 | 0.000 | 0.000 | 0.000 | 0.000 | 0.000 |  | 0.000 |  | **0.010** |
