# Supplementary material for: Evidence of Subdivisions on Evolutionary Timescales in a Large, Declining Marsupial Distributed across a Phylogeographic Barrier
Source: PLoS One. 2016 Oct 12;11(10):e0162789. doi: 10.1371/journal.pone.0162789 (PMC5061365; doi:10.1371/journal.pone.0162789)
Supplement: S2 Table — Significance of Wilcoxon paired-samples signed-ranks tests of genetic diversity measures tested between all pairs of 24 sampling sites of SHN wombats. (DOCX) [file pone.0162789.s004.docx]

**S2 Table.** **Comparisons among All Pairs of Sites.** Significance of Wilcoxon paired-samples signed-ranks tests of genetic diversity measures tested between all pairs of 24 sampling sites of SHN wombats: *H*_E_ (expected heterozygosity) below and AR (allelic richness) above diagonal. Because there were only 4 loci, the maximum P-value for pairwise tests between sites was 0.07, so these are taken as significant. Italics indicate sites from the extreme west (Nullarbor + FWC) or the extreme east (Murraylands) of the species’ distribution.

|  | 1 | 2 | 3 | 4 | 5 | 6 | 7 | 8 | 9 | 10 | 11 | 12 | 13 | 14 | 15 | 16 | 17 | 18 | 19 | 20 | 21 | 22 | 23 | 24 |
| --- | --- | --- | --- | --- | --- | --- | --- | --- | --- | --- | --- | --- | --- | --- | --- | --- | --- | --- | --- | --- | --- | --- | --- | --- |
| 1. Point Pearce | — | .72 | .72 | **.07** | **.07** | .46 | .72 | **.07** | .46 | **.07** | .27 | **.07** | .14 | **.07** | **.07** | **.07** | **.07** | **.07** | **.07** | **.07** | **.07** | **.07** | **.07** | **.07** |
| *2. Mannum* | .71 | — | .72 | .46 | .72 | 1.00 | 1.00 | .72 | .72 | .72 | .72 | .46 | .27 | .27 | .14 | **.07** | .14 | .14 | .14 | .14 | .14 | .14 | .14 | .14 |
| 3. MountWedge | .46 | .46 | — | .14 | .72 | .72 | .72 | **.07** | 1.00 | **.07** | .46 | .27 | **.07** | **.07** | .14 | **.07** | **.07** | **.07** | **.07** | **.07** | **.07** | **.07** | **.07** | **.07** |
| 4. Wallaroo | .46 | .46 | .46 | — | .27 | .46 | .46 | .72 | .72 | .72 | 1.00 | .72 | .46 | .14 | .46 | **.07** | **.07** | .14 | **.07** | **.07** | **.07** | **.07** | **.07** | **.07** |
| 5. Junkyard | .14 | .27 | 1.00 | .46 | — | 1.00 | .72 | .27 | .72 | .14 | .72 | .46 | .14 | **.07** | .14 | **.07** | .14 | .14 | **.07** | **.07** | **.07** | **.07** | **.07** | **.07** |
| 6. Scrubby Peak | .46 | .46 | 1.00 | 1.00 | .72 | — | 1.00 | .46 | .46 | .27 | .46 | **.07** | .28 | **.07** | **.07** | **.07** | .27 | .14 | **.07** | **.07** | **.07** | **.07** | .14 | **.07** |
| *7. Nullarbor* | .46 | .27 | .72 | 1.00 | .72 | .46 | — | .27 | .72 | .46 | .72 | .27 | .46 | .14 | **.07** | **.07** | .46 | .14 | **.07** | .14 | **.07** | **.07** | **.07** | **.07** |
| 8. Rickaby | **.07** | .46 | .72 | 1.00 | .46 | .72 | 1.00 | — | .46 | .72 | .46 | .72 | .46 | .72 | .72 | **.07** | .46 | 1.00 | .14 | .14 | .46 | .27 | .27 | .14 |
| 9. Lake Harris | .72 | .72 | .46 | .72 | .46 | .46 | .27 | .46 | — | .46 | .72 | .46 | .46 | .46 | .27 | **.07** | .46 | .27 | **.07** | .46 | .14 | **.07** | .27 | .14 |
| 1. Bramfield | **.07** | .27 | .46 | .72 | .72 | .72 | 1.00 | .72 | .46 | — | .72 | .72 | .72 | .11 | .46 | **.07** | .27 | .72 | **.07** | **.07** | .14 | **.07** | .46 | **.07** |
| *11. Eucla* | .27 | .46 | .46 | .72 | .72 | .46 | .72 | 1.00 | .72 | 1.00 | — | 1.00 | .27 | .46 | .46 | **.07** | .46 | .14 | **.07** | .14 | .14 | .14 | .14 | **.07** |
| 12. Rose Swamp | **.07** | .14 | .72 | .72 | .46 | .27 | .46 | .72 | .46 | .72 | .46 | — | .46 | .46 | **.07** | **.07** | .46 | .14 | **.07** | **.07** | **.07** | **.07** | .14 | **.07** |
| 13. Tiparra | .27 | .27 | .27 | .72 | .71 | .46 | 1.00 | .46 | .46 | 1.00 | 1.00 | 1.00 | — | 1.00 | .72 | **.07** | 1.00 | .46 | .46 | .46 | .72 | .46 | .46 | .14 |
| 14. Poochera | **.07** | .27 | **.07** | .14 | **.07** | .46 | .27 | .27 | .46 | .11 | .46 | .46 | .72 | — | 1.00 | .14 | 1.00 | 1.00 | .14 | .27 | .46 | .14 | .46 | .14 |
| *15. Fowler'sBay* | **.07** | .11 | .14 | .46 | .14 | .14 | .14 | .46 | .27 | .46 | .46 | **.07** | .72 | 1.00 | — | **.07** | 1.00 | 1.00 | .46 | 1.00 | .46 | .27 | 1.00 | .14 |
| *16. Ceduna* | **.07** | **.07** | .14 | .14 | .14 | .14 | .27 | .14 | .27 | .46 | .27 | .14 | .27 | .46 | .46 | — | **.07** | **.07** | .14 | **.07** | **.07** | .14 | **.07** | .14 |
| 17. Wauraltee | **.07** | .14 | **.07** | **.07** | .14 | .27 | .46 | **.07** | .46 | .27 | .27 | .46 | **.07** | .72 | 1.00 | .72 | — | 1.00 | .27 | .27 | .72 | .46 | .46 | .14 |
| 18. Kulpara | **.07** | **.07** | **.07** | **.07** | **.07** | .14 | **.07** | **.07** | .14 | .14 | .14 | .14 | .27 | .46 | .46 | .14 | .46 | — | .27 | .72 | 1.00 | .14 | .46 | .14 |
| *19. Nundroo* | **.07** | **.07** | **.07** | **.07** | **.07** | **.07** | .14 | .14 | .14 | **.07** | .14 | **.07** | .46 | .14 | .27 | .46 | .72 | 1.00 | — | .46 | .27 | .72 | .46 | .72 |
| *2. Sturt Highway* | **.07** | .14 | **.07** | **.07** | **.07** | .14 | .14 | **.07** | .46 | **.07** | .14 | .14 | .14 | **.07** | .72 | .46 | .46 | .72 | 1.00 | — | .72 | .46 | .72 | **.07** |
| *21. Coorabie* | **.07** | **.07** | **.07** | **.07** | **.07** | **.07** | **.07** | .27 | .14 | **.07** | .14 | **.07** | .27 | .14 | .46 | .72 | .72 | 1.00 | 1.00 | 1.00 | — | **.07** | .72 | .27 |
| 22. Hiltaba | **.07** | **.07** | **.07** | **.07** | **.07** | **.07** | **.07** | .14 | **.07** | **.07** | .14 | **.07** | .27 | .14 | .14 | .27 | .46 | .46 | .14 | .46 | **.07** | — | .27 | 1.00 |
| *23. Brookfield* | **.07** | **.07** | **.07** | **.07** | **.07** | .14 | **.07** | **.07** | .14 | **.07** | .14 | **.07** | **.07** | .14 | **.07** | **.07** | .46 | .14 | .27 | .27 | .46 | .72 | — | **.07** |
| *24. Swan Reach* | **.07** | **.07** | **.07** | **.07** | **.07** | **.07** | **.07** | **.07** | .14 | **.07** | **.07** | **.07** | **.07** | **.07** | **.07** | **.07** | .14 | .14 | **.07** | **.07** | .14 | .46 | .14 | — |

Sites rank-ordered by Friedman test from lowest *H*_E_ (Point Pearce, mean rank = 5.00) to highest *H*_E_ (Swan Reach, mean rank = 22.25).
