# Supplementary material for: Evidence of Subdivisions on Evolutionary Timescales in a Large, Declining Marsupial Distributed across a Phylogeographic Barrier
Source: PLoS One. 2016 Oct 12;11(10):e0162789. doi: 10.1371/journal.pone.0162789 (PMC5061365; doi:10.1371/journal.pone.0162789)
Supplement: S3 Table — Average probability of membership for individuals in six genetic clusters inferred by structure, using microsatellite data (four loci) from 24 L. latifrons populations. (DOCX) [file pone.0162789.s005.docx]

**S3 Table. Cluster Membership.** Average probability of membership for individuals in six genetic clusters inferred by structure, using microsatellite data (four loci) from 24 *L. latifrons* populations. Headings indicate geographic regions associated with each cluster and identify populations located in those regions. Bold numbers indicate the most highly associated cluster for each population.

|  |  |  | **Cluster** | | | | | |  |
| --- | --- | --- | --- | --- | --- | --- | --- | --- | --- |
| Site | Latitude | Longitude | 1 | 2 | 3 | 4 | 5 | 6 | *N* of individuals |
| **Nullarbor+ Far west coast (FWC)** | | | | | | | | | |
| Eucla | 31°40′S | 128°53′E | **0.949** | 0.015 | 0.010 | 0.010 | 0.008 | 0.008 | 9 |
| Nullarbor | 30°51′S | 130°28′E | **0.963** | 0.008 | 0.006 | 0.006 | 0.012 | 0.005 | 27 |
| Nundroo | 31°47′S | 132°12′E | **0.546** | 0.061 | 0.269 | 0.036 | 0.010 | 0.078 | 15 |
| Coorabie | 31°54′S | 132°18′E | **0.925** | 0.010 | 0.020 | 0.014 | 0.018 | 0.013 | 15 |
| Fowler’s Bay | 31°58′S | 132°34′E | **0.934** | 0.009 | 0.007 | 0.010 | 0.030 | 0.010 | 6 |
| Ceduna | 32°08′S | 133°41′E | **0.938** | 0.007 | 0.027 | 0.010 | 0.012 | 0.007 | 3 |
| **Gawler Ranges** | | | | | | | | | |
| Lake Harris | 31°04′S | 135°14′E | 0.031 | **0.900** | 0.013 | 0.037 | 0.010 | 0.009 | 14 |
| Rose Swamp | 31°17′S | 134°55′E | 0.013 | **0.962** | 0.008 | 0.005 | 0.006 | 0.007 | 9 |
| Hiltaba | 32°08′S | 135°03′E | 0.035 | **0.486** | 0.196 | 0.060 | 0.048 | 0.175 | 23 |
| Scrubby Peak | 32°31′S | 135°19′E | 0.008 | **0.955** | 0.011 | 0.006 | 0.012 | 0.007 | 24 |
| **Eyre Peninsula** | | | | | | | | | |
| Poochera | 32°43′S | 134°50′E | 0.276 | 0.019 | **0.592** | 0.054 | 0.027 | 0.033 | 7 |
| Bramfield | 33°38′S | 134°59′E | 0.032 | 0.112 | **0.732** | 0.055 | 0.018 | 0.051 | 10 |
| Mount Wedge | 33°29′S | 135°09′E | 0.103 | 0.007 | **0.859** | 0.012 | 0.009 | 0.010 | 10 |
| **South and west Yorke Peninsula** | | | | | | | | | |
| Rickaby | 34°43′S | 137°31′E | 0.007 | 0.005 | 0.010 | **0.807** | 0.164 | 0.007 | 5 |
| Wauraltee | 34°30′S | 137°36′E | 0.036 | 0.044 | 0.060 | **0.792** | 0.041 | 0.027 | 52 |
| Point Pearce | 34°24′ | 137°26′E | 0.008 | 0.006 | 0.008 | **0.958** | 0.013 | 0.007 | 7 |
| Junkyard | 34°25′ | 137°30′E | 0.010 | 0.007 | 0.015 | **0.922** | 0.027 | 0.019 | 7 |
| Wallaroo | 33°56′ | 137°36′E | 0.012 | 0.005 | 0.007 | **0.838** | 0.130 | 0.007 | 8 |
| **Northeast Yorke Peninsula** | | | | | | | | | |
| Kulpara | 34°04′S | 138°02′E | 0.041 | 0.022 | 0.034 | 0.019 | **0.853** | 0.031 | 89 |
| Tiparra | 34°06′S | 137°54′E | 0.013 | **0.508** | 0.029 | 0.028 | 0.347 | 0.074 | 7 |
| **Murraylands** | | | | | | | | | |
| Sturt Highway | 34°25′S | 139°08′E | 0.119 | 0.029 | **0.766** | 0.011 | 0.018 | 0.057 | 8 |
| Mannum | 34°54′S | 139°18′E | 0.007 | 0.007 | 0.475 | 0.006 | 0.011 | **0.494** | 4 |
| Swan Reach | 34°34′S | 139°36′E | 0.017 | 0.017 | 0.425 | 0.013 | 0.028 | **0.500** | 83 |
| Brookfield | 34°21′S | 139°24′E | 0.008 | 0.014 | 0.184 | 0.009 | 0.015 | **0.770** | 118 |
