# Supplementary material for: Evidence of Subdivisions on Evolutionary Timescales in a Large, Declining Marsupial Distributed across a Phylogeographic Barrier
Source: PLoS One. 2016 Oct 12;11(10):e0162789. doi: 10.1371/journal.pone.0162789 (PMC5061365; doi:10.1371/journal.pone.0162789)
Supplement: S1 Text — Chelex Extractions of Hair Samples. Phenol/IAC Extractions of Hair, Blood, and Tissue Samples. Amplification of Microsatellite Loci. Genotyping Process. (DOCX) [file pone.0162789.s006.docx]

**S1 Text. Supplementary Methods.**

*Chelex Extractions of Hair Samples*

Extractions were performed in the field to maximize DNA yield. Scissors and tweezers were ethanol flamed before handling individual hairs. The hair follicle and up to 1.5 cm of hair shaft was placed follicle-down in 200 *μ*l of 5% (w/v in 10mM Tris pH 8, 0.1mM EDTA pH 8) chelex® 100 resin (Bio-Rad) and boiled for 10 min. Extraction blanks (chelex but no hair) were always included. Chelex extracts were stored at 4°C for up to 2 weeks in the field, and longer term in the laboratory at −20°C.

*Phenol/IAC Extractions of Hair, Blood, and Tissue Samples*

Hairs with follicles were placed follicle-down in 500 *μ*l of ‘Higuchi’ extraction buffer (0.01M Tris, 0.01M EDTA, 0.1M NaCl, 50 *μ*g/ml Proteinase K [10mg/ml in distilled H_2_0], 0.039M DTT, 2% SDS, pH 8), and then incubated overnight at 45°C. Blood and tissue samples were extracted using DNA extraction buffer (0.15M NaCl, 0.1M EDTA, 20mM Tris-HCl, pH 8) plus 1% SDS and 100 *μ*g/ml Proteinase K, incubated overnight at 40°C. Each extraction was then placed in a 1.5 or 15ml centrifuge tube with an equal volume of buffer-saturated phenol (mixed 1:1 with TE [100mM Tris-HCl, 10mM EDTA], pH 7.5–8.5), mixed thoroughly and centrifuged at 10,000 g for 15 min. The top aqueous layer (containing the DNA) was collected and then re-extracted with phenol/IAC (chloroform and iso-amyl alcohol in the ratio 24:1 v/v) and then IAC alone. The DNA was precipitated by adding, to the final recovered aqueous layer, 1/10^th^ volume of 3M sodium acetate (pH 5.2) and two volumes of absolute ethanol (AnalaR) at −20°C and inverting the tube several times until the DNA clumped. The tube was then spun to pellet the DNA and the pellet washed in 1ml of 70% ethanol. The supernatant was removed and the pellet air dried at room temperature. The DNA was then suspended in 500ml 1X TE and stored at −20°C.

*Amplification of Microsatellite Loci*

The 13 loci (4 shared, 4 unique to set A, 5 unique to set B) were successfully amplified by the polymerase chain reaction (PCR) in a 10 *μ*l isotopic reaction containing 100–200 ng of template DNA, 0.5 units of *Taq* polymerase (Promega), 12 pmol of each primer, 200 *μ*M of dCTP, dGTP, and dTTP, 20 *μ*M of dATP, 2 mM MgCl_2_, 10 mM Tris-HCl (pH 8.3), 50 mM KCl, 0.1% Triton X-100 and 0.05 *μ*l [*α*-^33^P]dATP at 1000 Ci/mmol. Cycling was performed in a MJ Research PTC100 thermocycler, starting with 94°C for 2 min, followed by a ‘touchdown’ PCR (30 cycles of 94°C/15s, annealing/30s, 72°C/45s) and a final step of 72°C for 2 min. We used two different ‘touchdowns’: in ‘55→47’ the annealing temperature decreased two degrees per cycle, and the ‘62→55’ profile was 62-61-59-57-55°C. The following table identifies the touchdown used for each locus.

| **Locus** | **GenBank Accession No.** | **Touchdown** |
| --- | --- | --- |
| *Shared by sets A and B* | | |
| Lla54CA | AF185103 | 62→55 |
| Lla67CA | AF185105 | 55→47 |
| Lla68CA | AF185106 | 55→47 |
| Lla71CA | AF185107 | 55→47 |
| *Unique to set A* | | |
| Lla3AT | AF185101 | 55→47 |
| Lla16CA | AF185098 | 55→47 |
| Lla55A | AF185104 | 62→55 |
| Lkr107* | AF185113, Af185114 | 62→55 |
| *Unique to set B* | | |
| Ll2 | AF191296 | 62→55 |
| Lk13 | AF178639 | 55→47 |
| Lk21 | AF178641 | 55→47 |
| Lk23 | AF178642 | 62→55 |
| Lk37 | AF178649 | 62→55 |

*locus for which incomplete sequence data were obtained, requiring two separate accession numbers for its flanking sequences.

*Genotyping Process*

PCR products were run on 6% polyacrylamide sequencing gels next to A- or T-terminating M13 control sequencing reaction size markers, then visualized using autoradiography. For set B, positive biopsy controls from set A were used in addition to the M13 size markers, so that allele sizes could be compared across the two sets.
